# Every hit matters: White matter diffusivity changes in high school football athletes are correlated with repetitive head acceleration event exposure

**DOI:** 10.1016/j.nicl.2019.101930

**Published:** 2019-07-16

**Authors:** Ikbeom Jang, Il Yong Chun, Jared R. Brosch, Sumra Bari, Yukai Zou, Brian R. Cummiskey, Taylor A. Lee, Roy J. Lycke, Victoria N. Poole, Trey E. Shenk, Diana O. Svaldi, Gregory G. Tamer, Ulrike Dydak, Larry J. Leverenz, Eric A. Nauman, Thomas M. Talavage

**Affiliations:** a School of Electrical and Computer Engineering, Purdue University, West Lafayette, IN, United States of America; b Department of Neurology, Indiana University School of Medicine, Indianapolis, IN, United States of America; c Weldon School of Biomedical Engineering, Purdue University, West Lafayette, IN, United States of America; d College of Veterinary Medicine, Purdue University, West Lafayette, IN, United States of America; e School of Mechanical Engineering, Purdue University, West Lafayette, IN, United States of America; f School of Health Sciences, Purdue University, West Lafayette, IN, United States of America; g Department of Health and Kinesiology, Purdue University, West Lafayette, IN, United States of America; h Department of Basic Medical Sciences, Purdue University, West Lafayette, IN, United States of America

**Keywords:** Subconcussive injury, Traumatic brain injury, Magnetic resonance imaging, Diffusion-weighted imaging, Diffusion tensor imaging

## Abstract

Recent evidence of short-term alterations in brain physiology associated with repeated exposure to moderate intensity subconcussive head acceleration events (HAEs), prompts the question whether these alterations represent an underlying neural injury. A retrospective analysis combining counts of experienced HAEs and longitudinal diffusion-weighted imaging explored whether greater exposure to incident mechanical forces was associated with traditional diffusion-based measures of neural injury—reduced fractional anisotropy (FA) and increased mean diffusivity (MD). Brains of high school athletes (*N* = 61) participating in American football exhibited greater spatial extents (or volumes) experiencing substantial changes (increases and decreases) in both FA and MD than brains of peers who do not participate in collision-based sports (*N* = 15). Further, the spatial extents of the football athlete brain exhibiting traditional diffusion-based markers of neural injury were found to be significantly correlated with the cumulative exposure to HAEs having peak translational acceleration exceeding 20 g. This finding demonstrates that subconcussive HAEs induce low-level neurotrauma, with prolonged exposure producing greater accumulation of neural damage. The duration and extent of recovery associated with periods in which athletes do not experience subconcussive HAEs now represents a priority for future study, such that appropriate participation and training schedules may be developed to minimize the risk of long-term neurological dysfunction.

## Highlights

• Brain volumes evidencing injury are larger in football athletes than controls.
• Spatial extent of decreased FA correlates with head acceleration event exposure.
• Spatial extent of increased MD correlates with head acceleration event exposure.

## 1 Introduction

Researchers have observed that retired American football athletes (who have extended histories of exposure to subconcussive impacts) may have a higher risk of developing neurodegenerative disorders such as chronic traumatic encephalopathy, Alzheimer's disease, and Parkinson's disease (Omalu et al., 2006; Broglio et al., 2009; McKee et al., 2009; Stern et al., 2011; Lehman et al., 2012). Previous neuroimaging work has demonstrated changes in brain function and chemistry are associated with the accumulation of exposure to head acceleration events (HAEs), even in the absence of a diagnosis of concussion. Exposure to these “subconcussive” HAEs has been observed to be associated with alterations in the brain's response to task demands (Talavage et al., 2014; Robinson et al., 2015; Shenk et al., 2015), functional connectivity (Johnson et al., 2014; Abbas et al., 2015a, Abbas et al., 2015b), cerebrovascular reactivity (Svaldi et al., 2015, Svaldi et al., 2017, Svaldi et al., 2018), biochemical concentrations (Poole et al., 2014, Poole et al., 2015; Bari et al., 2018), and resting perfusion (Slobounov et al., 2017). Such alterations in function have been suggested as precursors to the symptoms normally resulting in the diagnosis of a concussion, with accumulation of HAEs put forth as a likely mechanism for symptom development (Bailes et al., 2013; Talavage et al., 2016).

While alterations in physiology like those reported above may arise from natural development or physical training (Lebel and Beaulieu, 2011; Lebel et al., 2012; Giorgio et al., 2010; Simmonds et al., 2014; Maddock et al., 2011), they may also arise from underlying structural damage to cells within the nervous system. Neural injury of this nature is typically assessed in MRI using diffusion-weighted imaging (DWI), with tensor-based analysis (diffusion tensor imaging, DTI) applied to focus on white matter integrity. In the healthy case, the DTI-measured diffusion of water molecules in white matter tracts is expected to be anisotropic—specifically, more directed along the length of an axon than outward through the cellular membrane and myelin sheath. Changes in the DTI-based measures of fractional anisotropy (FA) and the associated mean diffusivity (MD) are thus interpreted as markers or confirmation of changes to white matter health (e.g., Beaulieu, 2002; Arfanakis et al., 2002; Inglese et al., 2005; Bazarian et al., 2007).

For sport-related concussion and subconcussive trauma, measures of white matter health have been used to document changes associated with participation in a season of American football and soccer (Lipton et al., 2013; Bazarian et al., 2014; McAllister et al., 2014; Davenport et al., 2014, Davenport et al., 2016; Chun et al., 2015; Bahrami et al., 2016; Gong et al., 2018; Myer et al., 2018). In these studies, alterations in white matter health have been quantified through comparison of pre- and post-participation measures, with alterations in mean FA (or associated measures) interpreted as evidence of the effect of the intervening (and only sometimes quantified) HAEs. However, while these studies have revealed a number of changes in white matter health resulting from said participation, the results have been highly variable. Some studies have reported reduced mean FA and/or elevated mean MD (Arfanakis et al., 2002; Inglese et al., 2005; Miles et al., 2008; Cubon et al., 2011; Lipton et al., 2013; Mustafi et al., 2018), while others have provided evidence for increased mean FA and/or decreased mean MD (Bazarian et al., 2007, Bazarian et al., 2012; Wilde et al., 2008; Mayer et al., 2010, Mayer et al., 2012; Chu et al., 2010; Henry et al., 2011; Ling et al., 2012b; McAllister et al., 2012). Decreased FA and increased MD measurements are commonly considered to be indicative of impaired white matter structural integrity—e.g., myelin damage, including demyelination; or disruption of tissue structure, including axonal damage (Arfanakis et al., 2002; Kraus et al., 2007; Mac Donald et al., 2007a, Mac Donald et al., 2007b; Shitaka et al., 2011; Magnoni et al., 2015; Sundman et al., 2015; Pan et al., 2016; Kantarci et al., 2017). Conversely, increased FA and decreased MD are commonly attributed to either (a) axonal swelling arising from extra-neuronal injury, reducing space between fibers (Povlishock et al., 1983; Grady et al., 1993; Christman et al., 1994; Povlishock and Katz, 2005; Bazarian et al., 2012; Shultz et al., 2012; Johnson et al., 2013), or (b) cytotoxic edema and inflammation possibly resulting from ion homeostasis failure, membrane dysfunction, and microglial activation (Giza and Hovda, 2001; Hua Li et al., 2004; Giza and Prins, 2006; Marmarou et al., 2006; Wilde et al., 2008; Chu et al., 2010; Loane and Byrnes, 2010; Giza and Hovda, 2014; Giza et al., 2017).

Therefore, the critical question whether exposure to repeated HAEs produces what would be readily recognized as “injury” to the underlying brain structure remains open. Further the literature has not effectively addressed whether these reported changes—whether linked to injury or inflammation—are predominantly driven by natural growth, participation in intensive exercise, or are direct consequences of exposure to repeated subconcussive trauma. This retrospective study uses DTI acquired in a prospective study of high school-aged American football athletes to identify the nature and extent of the changes in white matter health and structure associated with the accumulation of exposure to HAEs. While assessments at the whole-brain level can reasonably be expected to reflect severe injuries, such as those associated with vehicular accidents or falls (e.g., Lipton et al., 2008, Lipton et al., 2013), the progression of damage to a symptom level likely requires a finer scale assessment. Confirmation that white matter alterations, likely to reflect one or both of inflammation or injury, are correlated with known mechanical exposures will provide key insight into the near-term risks of accumulation of repeated subconcussive events.

## 2 Methods

### 2.1 Participants

Previously-collected data from 181 high school-aged (i.e., ages 14–18) male athletes participating in American football (*N* = 162) or noncollision sports (*N* = 19) were evaluated for this study. Noncollision athletes indicated participation in track (*N* = 9), swimming (*N* = 6), cross-country (*N* = 7), or basketball (*N* = 2), with some participating in more than one sport. None of the included subjects reported having been diagnosed with a concussion within the 3 months prior to the period of study. Further, none of the athletes were diagnosed with a concussion by their team healthcare professionals during the period of study.

### 2.2 Participation schedule

#### 2.2.1 Football athletes (FBA)

**Fig. 1:**
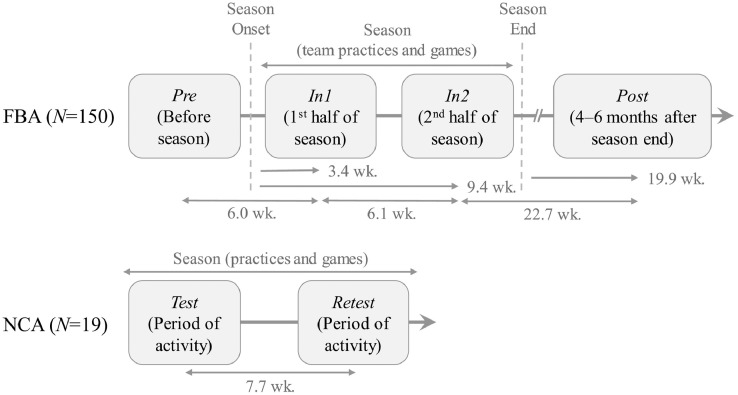
Participation schedule for football athletes (FBA) and peer non-collision-sport athletes (NCA). Average intervals are reported after rounding to the first decimal place.

150 of the 162 football athletes participated in at least four MRI sessions were scheduled to encompass one competition season: one in the 2 months preceding onset of contact practices (*Pre*); one each within the first (*In1*) and second (*In2*) 6-week segments of the competition season, corresponding to an average of 6 (*In1*) and 12 (*In2*) weeks after *Pre*; and one 4–6 months after the end of the competition season (*Post*), at an average of 35 weeks after *Pre* (see Fig. 1, top). Note that the *Pre* session took place during summer conditioning, when FBA were engaged in regular workouts involving light contact (e.g., “shells”), but did not involve full-contact (e.g., “thud”) or tackling practices. The average intervals (±standard deviation) between the onset of contact activities and the corresponding follow-up sessions were (*In1*) 3.4 ± 2.0 weeks; (*In2*) 9.4 ± 2.1 weeks; and (*Post*) 32.1 ± 2.4 weeks, corresponding to 19.9 ± 2.9 weeks after the cessation of contact activities.

#### 2.2.2 Noncollision-sport athletes (NCA)

The 19 noncollision-sport athletes were scanned twice (*Test* and *Retest*), at an interval of 5–18 weeks (average = 8 weeks), while actively engaged in training or competition, which are indistinguishable from one another in this population (see Fig. 1, bottom).

### 2.3 Head acceleration event monitoring

All football athletes were monitored for head acceleration events (HAEs) to assess relative mechanical loading across the population. Athletes were monitored throughout all team practices and games—for details, see Breedlove et al. (2012) and McCuen et al. (2015). Sensors used were either the HIT System (Simbex, LLC), a helmet-based telemetry system; or the xPatch (X2 Biosystems, Inc), a head-mounted sensor.

Both devices were set to record all events whose peak translational acceleration (PTA) exceeds 10 g, but analysis was conducted only on those events exceeding 20 g. Our previous work (Cummiskey et al., 2017) suggests that 20 g currently represents the lowest reasonable threshold for which the HIT System and xPatch are each reliable and consistent indicators of the presence of HAEs, as event counts exceeding this minimum threshold were found to be similar across both devices. It is critical to note that, based on laboratory testing, the (specific) magnitudes and locations provided for each HAE were not used in this work, as the errors associated with each individual measurement are substantial—sometimes exceeding 100% root-mean-square error—for these sensors (Jadischke et al., 2013; Cummiskey et al., 2017). However, given that both sensor systems have been found to be relatively unbiased on average (generally under 15% error; see Cummiskey et al., 2017), counts of events reported to exceed a given threshold may be used in a regression with increasing predictive power as the number of experienced events grows (i.e., the expected systematic undercounting will become more consistent across all subjects).

### 2.4 MRI data acquisition

All MRI sessions were performed at the Purdue MRI Facility, on a 3-T General Electric Signa HDxt (Waukesha, WI), using a 16-channel brain array (Nova Medical; Wilmington, MA). Head motion was minimized with restraining foam pads. DWI acquisitions used a two-dimensional single-shot spin-echo echo-planar imaging (EPI) sequence (repetition time [TR] = 12,000, echo time [TE] = 83.6 ms, flip angle = 90°, field of view = 240 mm × 240 mm, in-plane resolution = 2.5mm × 2.5mm, slice thickness = 2.5 mm, slice gap = 0 mm, 46 contiguous axial slices, frequency readout = R/L) with 30 diffusion encoding directions at *b* = 1000 s/mm^2^ and one volume acquired at *b* = 0 s/mm^2^. Raw images were upsampled to a 256 × 256 matrix by the MRI system for an image voxel size of 0.938 mm × 0.938 mm × 2.5 mm.

### 2.5 Data processing and quality assessment

#### 2.5.1 Pre-processing

DWI data were processed using FSL (Smith et al., 2004; Woolrich et al., 2009; Jenkinson et al., 2012). For each image, a brain mask was generated on the non-diffusion-weighted volume (i.e., *b* = 0) by segmenting brain from non-brain tissues (*BET*; Smith, 2002). Corrections were then applied for head movements and eddy current-induced distortions (*Eddy*; Andersson et al., 2016) while detecting slices with signal dropout and replacing them with Gaussian process predictions (−*repol* option in Eddy; Andersson and Sotiropoulos, 2016). Scalar diffusion tensor maps were then estimated by fitting the diffusion tensor model at each voxel (*FDT*; Behrens et al., 2003). Fractional anisotropy (FA) and mean diffusivity (MD) were subsequently calculated from the three primary eigenvalues.

#### 2.5.2 Quality assurance

Prior to voxel-wise analysis, quality assessment was performed on the images output from the preceding process, to ensure that acquisitions were not significantly corrupted. The following criteria for exclusion were similar to, but more stringent than, Murugavel et al. (2014). First, a computational assessment was conducted. Head movements during imaging were estimated 1) between every consecutively-acquired volumes and 2) relative to the first volume, based on each volume's registration parameters (Ling et al., 2012a). Subjects with at least one displacement relative to the first volume exceeding 5.0 mm were excluded from the study. For each scan, the average values of translations and rotations per unit time (i.e., time between two consecutive volumes within one DWI scan) were calculated across all 30 time points. Those subjects whose average movement exceeded three standard deviations (from the mean across all scans) in any translation/rotation along/around the x, y, or z-axis were also ruled out. Next a visual assessment was conducted, discarding any remaining data in which artifacts could be observed, or for which reconstruction had been improper.

After quality assessment, the resulting dataset comprised complete sets of data from 61 FBA (i.e., four valid imaging sessions and complete HAE data) and 15 NCA (i.e., both valid imaging sessions). See Table 1 for demographics of participants whose data passed screening and were included in analyses. The remaining 101 FBA were excluded for one or more of the following reasons: (a) they did not participate in all four imaging sessions, (b) they experienced an injury during the season that resulted in cessation of active participation, (c) their HAE data were incomplete (e.g., battery failure or sensor repeatedly fell off athlete), or (d) subject motion was excessive and the imaging data did not pass quality assurance. All 4 excluded NCA were on the basis of motion/failure to pass QA.

**Table 1:** Demographics of participants with complete set of valid imaging data.

	FBA (*N* = 61)	NCA (*N* = 15)
Age (years)		
Mean ± StdDev	16.6 ± 0.9	16.5 ± 1.2
[Min, Max]	[15, 18]	[14, 18]

Years of current sport (high school)		
Mean ± StdDev	2.2 ± 0.8	2.1 ± 1.0
[Min, Max]	[0, 3]	[0, 3]

Number of previously diagnosed concussions		
Mean ± StdDev	0.6 ± 1.0	0.5 ± 0.8
[Min, Max]	[0, 5]	[0, 2]

Racial and ethnic categories		
White	43	13
Black or African American	14	0
Hispanic or Latino	1	0
Asian	1	2
More than one	2	0

#### 2.5.3 Image registration

Datasets that passed quality assessment were input to a subset of the Tract-Based Spatial Statistics (TBSS) pipeline (Smith et al., 2004, Smith et al., 2006; Andersson et al., 2007a,b) in FSL, to obtain and assess predominant fiber tracts within white matter of the brain. All FA images were nonlinearly registered to the 1 mm isotropic FMRIB58-FA standard-space image, yielding a transformation for each subject. Consistent with prior studies (e.g., Kraus et al., 2007; Oni et al., 2010; Gajawelli et al., 2013; Myer et al., 2016b; Slobounov et al., 2017; Kuzminski et al., 2018), a mean FA image was calculated from the registered images from all subjects, and thresholded at 0.2 to create a mean white matter (WM) skeleton, hereafter referred to as ROI_WM_.

Subsequently, the aligned FA image of the *i-th* subject obtained at the *j-th* session was projected onto ROI_WM_ to form an FA skeleton specific to each session and individual (FA_WM,*j,i*_). MD skeletons (MD_WM,*j,i*_) were similarly created by applying the subject's transformation to the raw MD images followed by projection to ROI_WM_.

### 2.6 Statistical analysis

Standard methodologies as described in Glantz (2012) were applied throughout for statistical testing purposes.

#### 2.6.1 Statistical tools

Statistical analyses were performed using STATA 14.0 (StataCorp LP; College Station, TX), MATLAB R2016 (MathWorks), and FMRIB Software Library (FSL) 5.0.

#### 2.6.2 Repeated measures testing

In cases of analyses across sessions and groups, the Shapiro-Wilk test for normality and Bartlett test of sphericity were conducted to ensure the validity of using a one-way repeated measures analysis of variance (ANOVA). If the normality assumption was violated, the Friedman non-parametric test was used in its place. If the sphericity assumption was violated, the Huynh-Feldt correction was applied to the resulting statistics.

#### 2.6.3 Two-sample hypothesis testing

For comparison across two sets of data, the Shapiro-Wilk test for normality was conducted to ensure the validity of using a *t*-test. If this normality assumption was violated, the non-parametric Wilcoxon rank-sum test (also called the Mann-Whitney *U* test) was used.

### 2.7 Image analysis

Several analyses were conducted to detect and evaluate the dependence of changes in white matter diffusion on exposure to the repetitive subconcussive HAEs (associated with a single competition season of American football). First, group-level longitudinal changes were sought by analyzing the magnitude and spatial extent (effectively a volume, comprising a collection of voxels) of changes in FA and MD. Second, the spatial extents of substantial changes in FA or MD (“change masks”) were identified at the individual subject level—noting that white matter locations at which FA or MD decreased and increased were identified separately, given these alterations have different pathophysiologic implications. Third, correlations between the spatial extents of the aforementioned change masks and the accumulated HAE exposure were analyzed.

#### 2.7.1 Group-level longitudinal changes

##### 2.7.1.1 Longitudinal changes in mean FA/MD

Consistent with prior literature (e.g., Meier et al., 2015; Henry et al., 2011; McAllister et al., 2014; Myer et al., 2016a), one-way repeated measures ANOVAs were conducted to determine if either the NCA or FBA groups exhibited longitudinal changes across sessions in mean FA ($\Delta {\bar{\mathrm{FA}}}_{\mathrm{WM}}$) or mean MD ($\Delta {\bar{\mathrm{MD}}}_{\mathrm{WM}}$), as averaged over the entire white matter skeleton (i.e., ROI_WM_)—i.e., ${\bar{\mathrm{FA}}}_{\mathrm{WM},j,i}$ and ${\bar{\mathrm{MD}}}_{\mathrm{WM},j,i}$, where for *i* ∈ FBA: *j* ∈ {*Pre*, *In*1, *In*2, *Post*}; and for *i* ∈ NCA: *j* ∈ {*Test*, *Retest*}. A further one-way ANOVA was performed to assess whether race had a significant effect on mean FA or mean MD.

Given the hypothesis that FBA who are no longer exposed to repeated HAEs might exhibit natural recovery toward baseline values of FA and MD (e.g., after the competition season ends), a second set of repeated measures ANOVAs was conducted on the mean FA and mean MD for this population to evaluate changes, relative to *Pre*, only for those follow-up sessions acquired during the active accrual of HAEs (i.e., *In1, In2*).

When significant effects of session were observed, pairwise comparison post hoc tests were conducted on the (subject-level) values of mean FA or mean MD in the corresponding population to identify those sessions that significantly differed in mean from baseline (i.e., estimated marginal mean).

#### 2.7.2 Subject-level longitudinal changes

Individual subject changes during and after exposure to HAEs were obtained through subject-specific quantification of extent of volumetric masks comprising voxels in which FA or MD values were significantly-altered over time.

##### 2.7.2.1 Individual subject mask generation

Given that we might expect changes in FA (or MD) over time in adolescent athletes (Giorgio et al., 2010; Lebel and Beaulieu, 2011; Lebel et al., 2012; Simmonds et al., 2014), it is desirable to further refine our analysis to focus only on those voxels in which we observe across-session changes unlikely to be associated with normal white matter development. Masks of change in FA (ΔFA) or MD (ΔMD) were generated using the population statistics from the NCA as a reference, assuming that changes markedly outside the range observed in the NCA population are potentially a consequence of exposure to repeated HAEs.

**Fig. 2:**
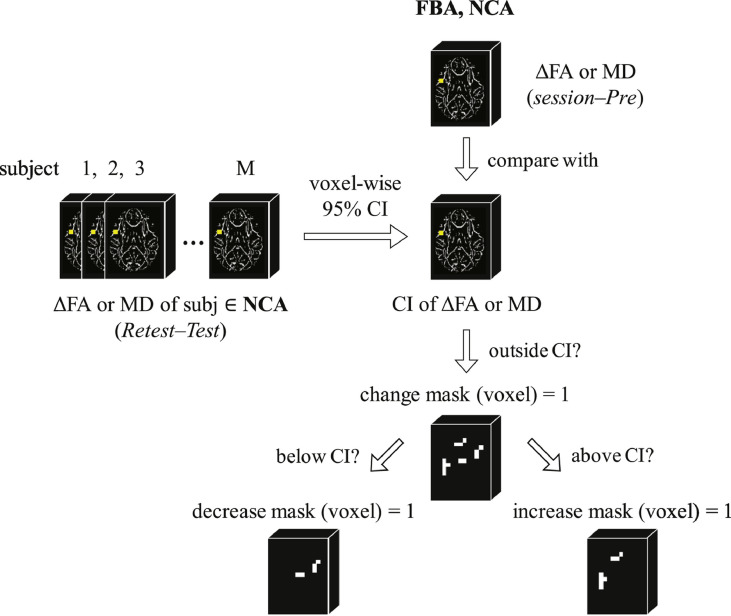
Illustration of the process by which FA and MD change masks (unsigned and signed) were generated at each imaging session for each member of the FBA and NCA populations, in both the white matter skeleton (ROI_WM_) and in the altered white matter mask (ROI_ALT_). See text (*Individual subject mask generation*) for details.

To this end, “change masks” that reflected any substantial alteration in one of FA or MD at a follow-up session, relative to baseline, were constructed for all subjects (both NCA and FBA) as follows. First, for each voxel in ROI_WM_ a 95% confidence interval (CI) was constructed from the NCA pool based on FA (or MD) changes observed at *Retest* relative to *Test*. Second, a change mask was created for each athlete (FBA and NCA) at each follow-up session (FBA: *In1, In2*, and *Post*; NCA: *Retest*), through identification of those voxels for which the subject's change in FA (or MD), relative to *Pre*, fell outside the corresponding voxel-specific NCA-defined 95% CI. Note that voxels for which the observed change in FA (or MD) fell outside the 99.9% CI are likely to be erroneous, and were excluded when creating subject-specific masks. (See Fig. 2.) The resulting (unsigned) change masks (roi_ΔFA_WM,*j,i*_, roi_ΔMD_WM,*j,i*_) represent the spatial extents of substantial change for the corresponding measures for subject *i* at follow-up session *j*.

Given the potentially different pathophysiologic causes of increases and decreases in FA and MD, separate (signed) change masks were generated for each direction of substantial alteration. An “increase change mask” was generated for each FBA and NCA subject, at each follow-up session, by identifying the subset of FA (or MD) change mask voxels exhibiting changes from *Pre* exceeding the upper 95% CI bound ($\text{roi}\_\Delta {\text{FA}}_{\text{WM},j,i}^{+}$, $\text{roi}\_\Delta {\text{MD}}_{\text{WM},j,i}^{+}$). Similarly, a “decrease change mask” was generated for each FBA and NCA subject, at each follow-up session, by identifying the subset of FA (or MD) change mask voxels exhibiting changes from *Pre* falling below the lower 95% CI bound ($\text{roi}\_\Delta {\text{FA}}_{\text{WM},j,i}^{-}$, $\text{roi}\_\Delta {\text{MD}}_{\text{WM},j,i}^{-}$).

##### 2.7.2.2 Comparison of change mask spatial extents between FBA and NCA

For each of the six change masks (unsigned, increase, decrease; FA and MD) created for each subject above, the spatial extent of the change mask, as a percentage of the total voxel count in ROI_WM_, was computed and compared across the FBA and NCA pools. These spatial extents are indicated as ‖ ⋅ ‖ operating on a given change mask (i.e., ‖$\text{roi}\_\Delta {\text{MD}}_{\text{WM},j,i}^{-}$‖ indicates the percentage extent, relative to the white matter skeleton, of the signed change mask associated with substantial decreases in MD).

##### 2.7.2.3 Correlation of longitudinal changes in FBA masks with HAE exposure

For all FBA subject change masks in ROI_WM_, Pearson (linear relationship) and Spearman (monotonic relationship) correlation analyses were conducted across each FBA follow-up session (*In1, In2, Post*), comparing the spatial extent of the change mask with the number of HAEs experienced to-date in practices and games that exceeded a given PTA threshold. PTA thresholds examined were 20 g, 30 g, 40 g, 50 g, 60 g, and 70 g. The upper-bound threshold of 70 g was selected as it represents approximately the 90th percentile of all recorded HAEs (Bari et al., 2018), and was expected to preserve a sufficient number of events per athlete such that the count remained accurate across subjects (cf. Cummiskey et al., 2017). Thresholds at which the correlation met a corrected significance level of *p*_Bonferroni_ < 0.05 were noted.

At the PTA threshold exhibiting the most significant correlation, a linear regression analysis was conducted to characterize the relationship between the change mask spatial extents at each FBA follow-up session and the number of HAEs experienced to-date, exceeding said threshold. For this regression, the confidence intervals for the true regression lines (i.e., confidence bands) were determined. Regressions for which the confidence bands did not contain a slope of zero were interpreted to be suggestive of a contribution to white matter alterations from exposure to repeated (sub-concussive) HAEs exceeding the identified threshold.

##### 2.7.2.4 Group-level altered WM mask

**Fig. 3:**
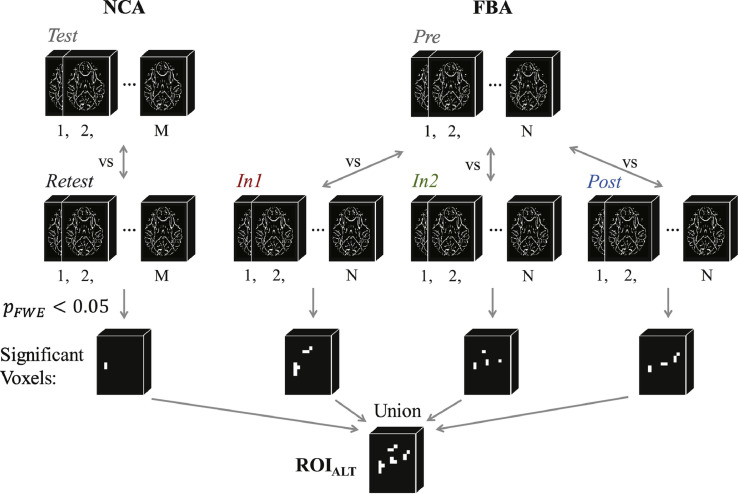
Illustration of the process by which the altered white matter mask, ROI_ALT_, was generated. Pair-wise (by session) permutation *t*-tests (50,000 permutations) were conducted for all follow-up sessions (NCA: *Retest*; FBA: *In1, In2, Post*) with baseline (NCA: *Test*; FBA: *Pre*) to identify voxels exhibiting a statistically-significant change (*p*_FWE_ < 0.05) at that follow-up session. The resulting sets of voxels for each follow-up session were merged to produce the altered white matter mask. See text (*Group-level altered WM mask*) for details.

Based on the outcome of the regression analysis for the entire white matter skeleton, we desired to assess the degree to which exposure to repeated HAEs was driving the observation of statistically-significant changes in white matter measures. Therefore, the ensemble of voxels exhibiting a statistically-significant change, relative to baseline (FBA: *Pre*; NCA: *Test*) at any follow-up session (FBA: *In1, In2*, or *Post*; NCA: *Retest*) was identified through use of a pair-wise (by session) permutation *t*-test (50,000 permutations for each of *Pre* vs. *In1, Pre* vs. *In2, Pre* vs. *Post, Test* vs. *Retest*). All results of the permutation *t*-test underwent threshold-free cluster enhancement (Smith and Nichols, 2009) and multiple-comparison correction for family-wise error (FWE), and voxels exhibiting *p*_FWE_ < 0.05 were identified at each follow-up session. The resulting voxels were combined across tests to produce a mask of altered white matter, ROI_ALT_. In other words, ROI_ALT_ represents the subset of voxels within ROI_WM_ that exhibited any significant change (increase or decrease) relative to baseline, at any follow-up session (see Fig. 3).

For the purpose of understanding the spatial distribution of the voxels found to exhibit statistically-significant changes in any follow-up session, the WM tracts containing voxels within ROI_ALT_ were identified through matching with the digital white matter atlas from Johns Hopkins University (JHU ICBM-DTI-81; Mori et al., 2005).

##### 2.7.2.5 Subject-level longitudinal changes in altered WM mask

Using the same methods as described above, six change masks (unsigned: roi_ΔFA_ALT,*j,i*_, roi_ΔMD_ALT,*j,i*_; signed: $\text{roi}\_\Delta {\text{FA}}_{\text{ALT},j,i}^{+}$, $\text{roi}\_\Delta {\text{FA}}_{\text{ALT},j,i}^{-}$, $\text{roi}\_\Delta {\text{MD}}_{\text{ALT},j,i}^{+}$, $\text{roi}\_\Delta {\text{MD}}_{\text{ALT},j,i}^{-}$) were generated from the voxels comprising ROI_ALT_. Note that this process is identical to the intersection of ROI_ALT_ with each of the subject change masks generated earlier (e.g., roi_ΔMD_ALT,*j,i*_ = roi_ΔMD_WM,*j,i*_ ∩ ROI_ALT_). The resulting change masks were evaluated using the same methodologies as above (for ROI_WM_), assessing (1) longitudinal changes in mean FA and mean MD within ROI_ALT_, (2) group differences, between FBA and NCA, in FA and MD change mask spatial extents within ROI_ALT_, and (3) the longitudinal relationship between FBA change mask spatial extents within ROI_ALT_ and HAE exposure.

## 3 Results

### 3.1 HAE exposures

**Fig. 4:**
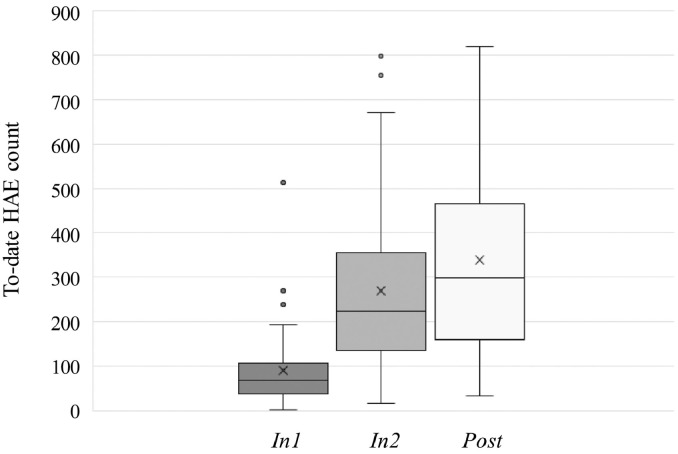
Distributions of “to-date” HAE exposures exceeding 20 *g* as of each follow-up session for the 61 FBA subjects.

Fig. 4 summarizes the distribution (median, 1st and 3rd quartile) of counts of HAEs experienced by the corpus of 61 FBA in practices and games, from the beginning of the scholastic season to the time of the corresponding follow-up sessions (*In1, In2, Post*). A repeated measures ANOVA revealed no effect of race (categories: *White, Black/African American, Hispanic/Latino, Asian, More than one*)—the only demographic factor that differed across the NCA and FBA groups—on to-date HAEs at each follow-up session.

### 3.2 Group-level longitudinal changes

#### 3.2.1 Longitudinal changes in mean FA/MD

**Fig. 5:**
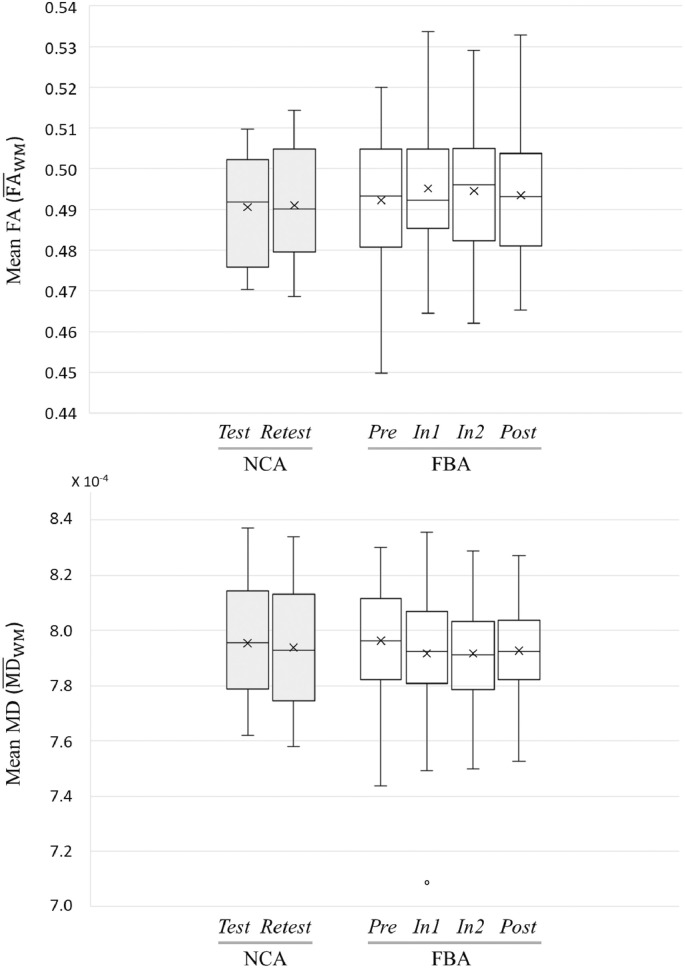
Box-and-whisker plots of distributions of (*top*) mean FA and (*bottom*) mean MD over the white matter skeleton (ROI_WM_) as a function of session for FBA (*N* = 61) and NCA (*N* = 15). The horizontal line in each box indicates the median and × indicates the mean. Note that the single outlier subject-session observed for mean MD in FBA at follow-up session *In1* was excluded from subsequent ANOVA-based tests.

Population distributions over the white matter skeleton (ROI_WM_) of mean FA (${\bar{\mathrm{FA}}}_{\mathrm{WM}}$) and mean MD (${\bar{\mathrm{MD}}}_{\mathrm{WM}}$) are presented as a function of group and session in Fig. 5. Note that at baseline, there was no significant difference in either mean FA or mean MD between FBA and NCA. Further, as with HAEs, above, a repeated measures ANOVA revealed no effect of race or age on mean FA or mean MD across sessions.

As seen in Table 2, there was no effect on session for NCA or FBA when all available (baseline plus follow-ups) were evaluated by repeated measures ANOVA. However, the repeated measures ANOVA conducted on FBA using only those follow-up sessions acquired during period of exposure to HAEs (i.e., *Pre, In1*, and *In2*) revealed a statistically-significant effect of session for mean FA. Post hoc pairwise analysis of the three FBA sessions revealed significant changes in mean FA relative to *Pre*, at both *In1* and *In2* (Table 3). Note that in Table 2 one session (at *In1*) of one FBA subject was excluded as an outlier from the repeated measures ANOVA for mean MD, because the subject's ${\bar{\mathrm{MD}}}_{\mathrm{WM},\mathrm{In}1}$ value was more than 250% of the interquartile range below the 25th percentile (see Fig. 5, bottom).

**Table 2:** Repeated measures ANOVA outcomes evaluating mean FA and mean MD in ROI_WM_ across indicated sessions.

Group	FA/MD	Sessions	*F*-statistic	Corrected-*p*
NCA	${\bar{\mathrm{FA}}}_{\mathrm{WM}}$	All sessions	F(1, 14) = 0.08	0.785
	${\bar{\mathrm{MD}}}_{\mathrm{WM}}$	All sessions	F(1, 14) = 0.23	0.642
FBA	${\bar{\mathrm{FA}}}_{\mathrm{WM}}$	All sessions	F(3, 180) = 2.44	0.140
	${\bar{\mathrm{MD}}}_{\mathrm{WM}}$	All sessions	F(3, 179) = 2.59	0.114
	${\bar{\mathrm{FA}}}_{\mathrm{WM}}$	*Pre, In1, In2*	F(2, 120) = 3.92	**0.044**⁎
	${\bar{\mathrm{MD}}}_{\mathrm{WM}}$	*Pre, In1, In2*	F(2, 119) = 3.51	0.066

Where the sphericity assumption was violated, *p*-values were corrected with Huynh–Feldt's method. Final *p*-values were corrected using a Bonferroni correction, given FBA data underwent two tests.

⁎ indicates a result significant at the (corrected) p < 0.05 level.

**Table 3:** Post hoc pairwise *t*-test comparisons of mean FA measurements in ROI_WM_ of FBA, acquired in sessions associated with accumulation of HAEs, for which a significant effect of session was observed (see Table 2).

Pairwise comparisons	*t*-Statistic	Corrected-*p*
*In1* vs. *Pre*	2.64	**0.013**⁎
*In2* vs. *Pre*	2.13	**0.035**⁎
*In2* vs. *In1*	−0.51	0.611

Duncan's method was used to correct for multiple comparisons. (Note that a positive sign for *t* indicates that the measurement in second listed session is greater than the measurement in the first.)

⁎ Indicates a result significant at the (corrected) p < 0.05 level.

**Fig. 6:**
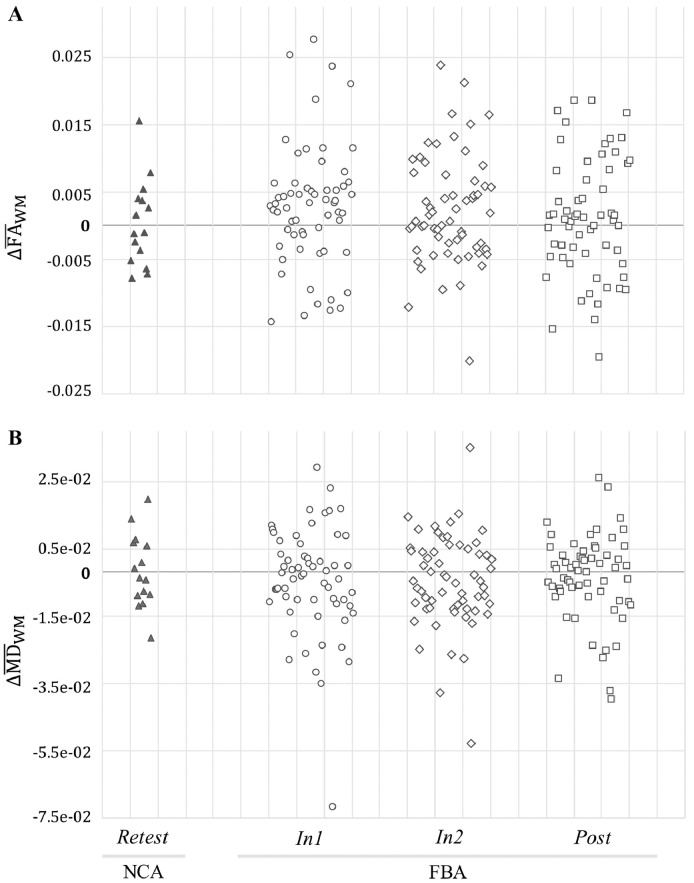
Changes in mean FA and mean MD in ROI_WM_ for FBA (*N* = 61) and NCA (*N* = 15) at each follow-up session compared to baseline (*Pre* for FBA; *Test* for NCA). FBA exhibited a greater spread of change values at each follow-up session (*In1, In2, Post*) than did NCA at *Retest*.

Scatter plots of changes, relative to baseline, in mean FA and mean MD for all FBA and NCA subjects at each follow-up session (i.e., $\Delta {\bar{\mathrm{FA}}}_{\mathrm{WM},j,i}$, $\Delta {\bar{\mathrm{MD}}}_{\mathrm{WM},j,i}$) are shown in Fig. 6. It may be readily observed that FBA consistently exhibit broader distributions of changes (both increases and decreases) than do NCA.

### 3.3 Subject-level longitudinal changes

#### 3.3.1 Comparison of change mask spatial extents between FBA and NCA

**Fig. 7:**
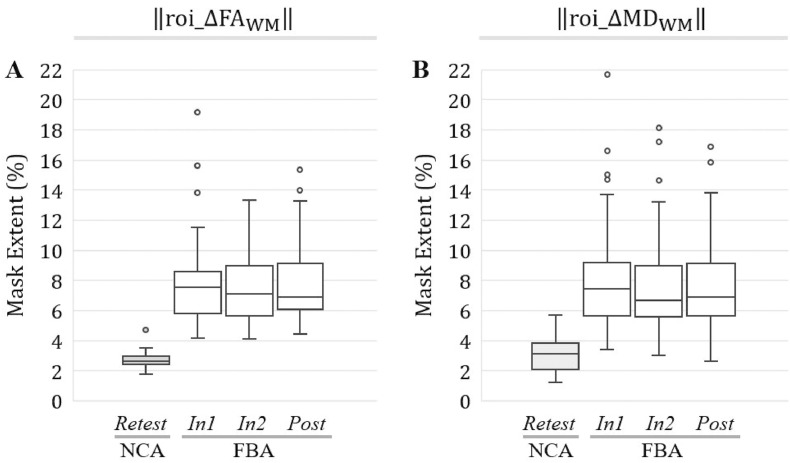
At all follow-up sessions, football athletes (FBA) exhibited significantly (*p* < 0.001) greater spatial extents of substantial changes in FA and MD, in ROI_WM_, than did noncollision athletes (NCA). Box-and-whisker plots are presented at each follow-up session for (A) ‖roi_ΔFA_WM,*j,i*_‖; (B) ‖roi_ΔMD_WM, *j,i*_‖.

For the entirety of the white matter skeleton (ROI_WM_) the spatial extents of substantial changes in both FA and MD—i.e., ‖roi_ΔFA_WM,*j,i*_‖ and ‖roi_ΔMD_WM,*j,i*_‖—were significantly larger (*p* < 0.0001; *t*-test or Wilcoxon rank-sum test) at each follow-up session for FBA relative to NCA (Fig. 7).

**Fig. 8:**
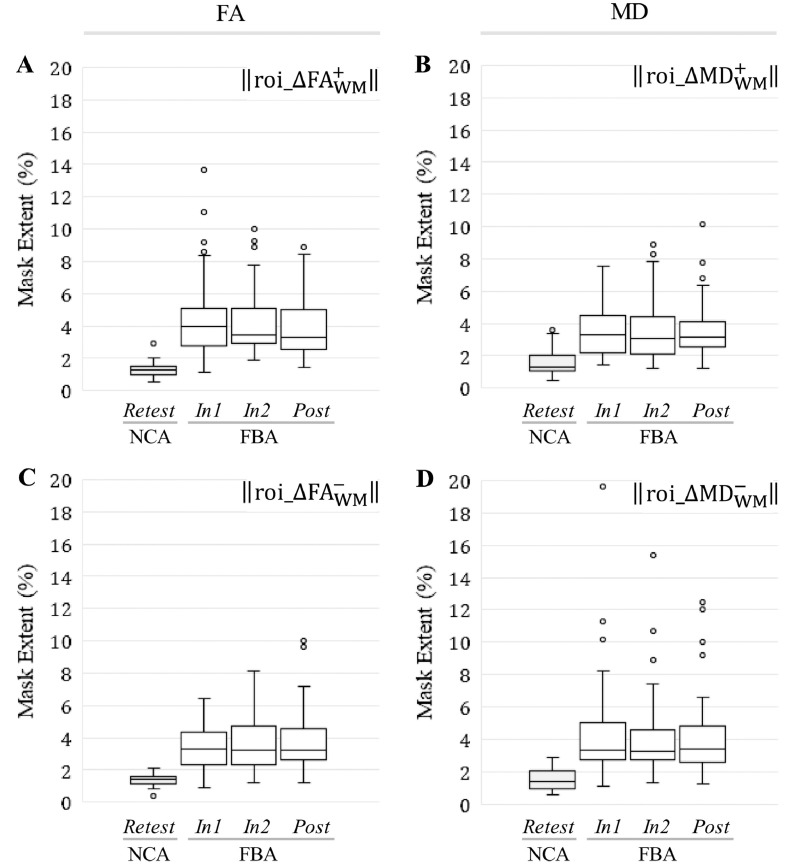
At all follow-up sessions, football athletes (FBA) exhibited significantly (*p* < 0.001) greater spatial extents of substantial increase/decrease in FA and MD, within ROI_WM_, than did noncollision athletes (NCA). Box-and-whisker plots are presented at each follow-up session for (A) ‖$\text{roi}\_\Delta {\text{FA}}_{\text{WM},j,i}^{+}$‖; (B) ‖$\text{roi}\_\Delta {\text{MD}}_{\text{WM},j,i}^{+}$‖; (C) ‖$\text{roi}\_\Delta {\text{FA}}_{\text{WM},j,i}^{-}$‖; (D) ‖$\text{roi}\_\Delta {\text{MD}}_{\text{WM},j,i}^{-}$‖.

Consistent with the differences observed for the unsigned change masks, all signed change masks associated with ROI_WM_ were also found to be significantly larger in spatial extent (*p* < 0.001; *t*-test or Wilcoxon rank-sum test) at each follow-up session for FBA relative to NCA (Fig. 8).

#### 3.3.2 Correlation of longitudinal changes in FBA masks with HAE exposure

To-date HAE accumulations were only found to be significantly correlated with the spatial extent of the change mask reflecting substantial decreases in FA (i.e., ‖$\text{roi}\_\Delta {\text{FA}}_{\text{WM}}^{-}$‖), and only for PTA thresholds of 20 g and 30 g (see Table 4).

**Table 4:** Pearson's and Spearman's correlation coefficients relating cumulative HAE counts exceeding the indicated minimum linear acceleration threshold (20–70 g), across all follow-up imaging sessions (i.e., *In1, In2, Post*; using the end-of-season HAE count for *Post*), to the signed change mask spatial extents in ROI_WM_.

ROI extent	‖$\text{roi}\_\Delta {\text{FA}}_{\text{WM}}^{+}$‖		‖$\text{roi}\_\Delta {\text{FA}}_{\text{WM}}^{-}$‖		‖$\text{roi}\_\Delta {\text{MD}}_{\text{WM}}^{+}$‖		‖$\text{roi}\_\Delta {\text{MD}}_{\text{WM}}^{-}$‖	
Included HAEs	Pearson	Spearman	Pearson	Spearman	Pearson	Spearman	Pearson	Spearman
PLA > 20 g	0.096	0.087	**0.231**⁎	0.171	0.170	0.158	0.089	0.003
PLA > 30 g	0.100	0.069	**0.201**⁎	0.154	0.130	0.138	0.111	−0.015
PLA > 40 g	0.125	0.074	0.164	0.142	0.096	0.130	0.143	−0.013
PLA > 50 g	0.122	0.058	0.122	0.109	0.057	0.091	0.127	−0.021
PLA > 60 g	0.130	0.082	0.085	0.064	0.026	0.040	0.121	0.012
PLA > 70 g	0.131	0.103	0.102	0.113	0.033	0.076	0.113	0.018

⁎ Correlation significant at the *p*_Bonferroni_ < 0.05 level.

**Fig. 9:**
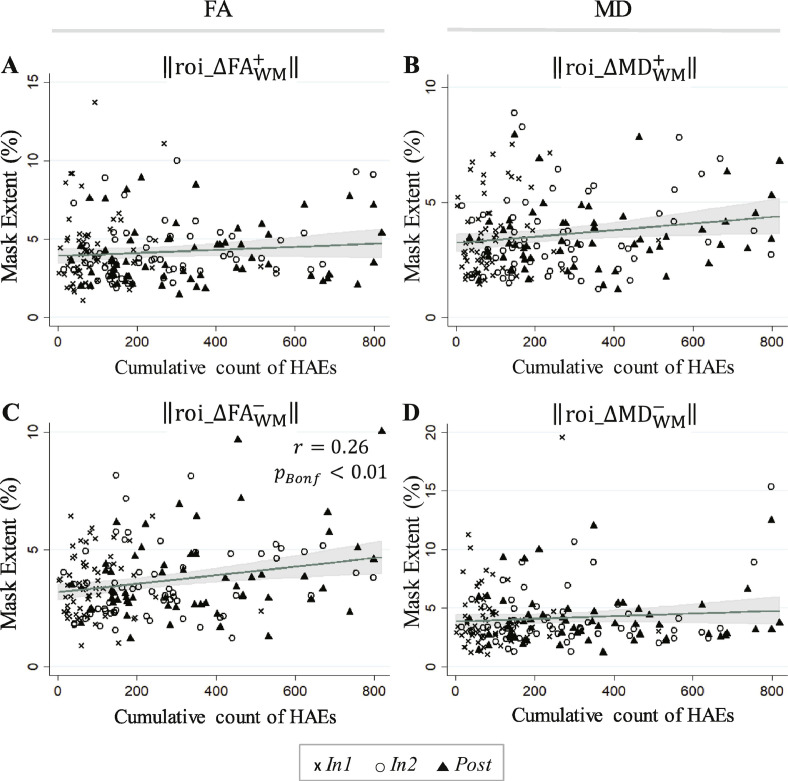
Linear predictions and associated confidence bands are superimposed on 183 FBA samples (61 subjects at each of three follow-up sessions) for (A) ‖$\text{roi}\_\Delta {\text{FA}}_{\text{WM}}^{+}$‖; (B) ‖$\text{roi}\_\Delta {\text{MD}}_{\text{WM}}^{+}$‖; (C) ‖$\text{roi}\_\Delta {\text{FA}}_{\text{WM}}^{-}$‖; (D) ‖$\text{roi}\_\Delta {\text{MD}}_{\text{WM}}^{-}$‖. A significant linear regression (Bonferroni-corrected; see Table 4) was found in FBA for the spatial extent of the ROI_WM_ signed change mask associated with a decrease in FA ($\text{roi}\_\Delta {\text{FA}}_{\text{WM}}^{-}$), as a function of the cumulative count of HAEs exceeding 20 g. This white matter change (decreased FA) is typically associated with neural injury (e.g., Wieshmann et al., 1999, Arfanakis et al., 2002, Kraus et al., 2007, Mac Donald et al., 2007a, Mac Donald et al., 2007b; Mac Donald et al., 2007a, Mac Donald et al., 2007b; Shitaka et al., 2011, Magnoni et al., 2015, Sundman et al., 2015, Pan et al., 2016, Kantarci et al., 2017). The symbol *r* represents a Pearson's correlation coefficient.

Regressions against the HAE count at 20 g, the threshold associated with the most significant correlation with signed change mask spatial extents, are shown for all signed change masks in Fig. 9. Consistent with Table 4, the regression fit for ‖$\text{roi}\_\Delta {\text{FA}}_{\text{WM}}^{-}$‖ against the to-date accumulation of HAEs exceeding 20 g was found to be statistically significant.

#### 3.3.3 Group-level altered WM mask

**Fig. 10:**
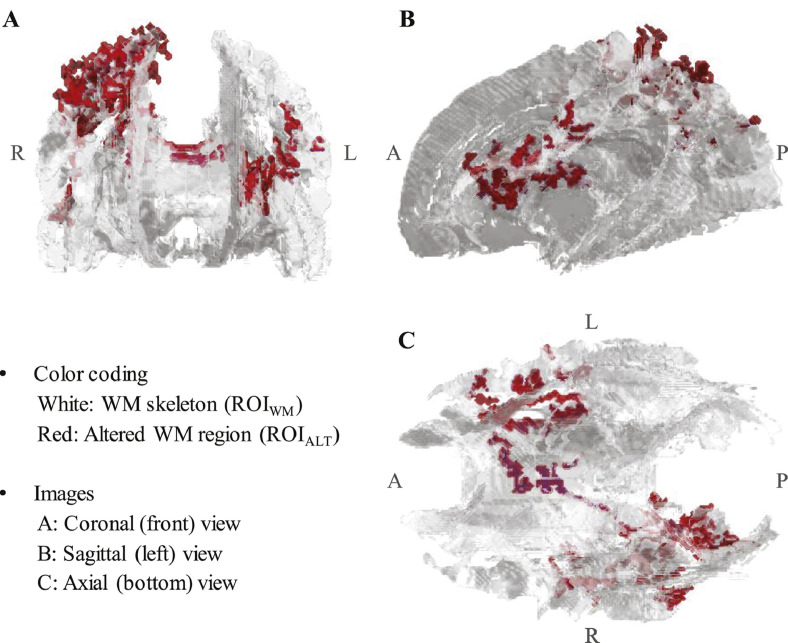
3D visualization (MATLAB) of ROI_ALT_ depicted on a fractional anisotropy-derived white matter skeleton, ROI_WM_. 3.96% (comprising 14 WM tracts) of the tested volume was found to exhibit significantly greater/lesser fractional anisotropy (FA) at one or more follow-up session (FBA: *In1, In2, Post*; NCA: *Retest*), relative to baseline (FBA: *Pre*; NCA: *Test*). White matter tracts in which ROI_ALT_ voxels were observed are listed in Table 5.

The mask comprising white matter skeleton voxels exhibiting significant alterations (*p*_FWE_ < 0.05; permutation *t*-test with 50,000 iterations) relative to FBA and/or NCA baseline, ROI_ALT_, is depicted via three-view projection in Fig. 10. This group-level region represents 3.96% of the white matter skeleton, comprising 4398 of the 110,939 (interpolated) voxels in ROI_WM_. Note that no voxels were found to be significantly changed in FA from *Test* to *Retest* in NCA. The resulting altered white matter mask, ROI_ALT_, intersects with 14 of the WM tracts defined in the JHU ICBM-DTI-81 atlas (Mori et al., 2005), as indicated in Table 5.

**Table 5:** White matter tracts (JHU ICBM-DTI-81 atlas) in which voxels from ROI_ALT_, shown in Fig. 10, were located. The percentage of voxels comprising ROI_ALT_ that were located in each tract is indicated. (The total may not sum to 100% due to rounding.)

White matter tract	Proportion of ROI_ALT_	JHU WM atlas labels
Corpus callosum	27.1%	Body of corpus callosum
		Splenium of corpus callosum
		Genu of corpus callosum
Superior longitudinal fasciculus	26.0%	Superior longitudinal fasciculus R
		Superior longitudinal fasciculus L
Limb of internal capsule	17.8%	Anterior limb of internal capsule L
		Posterior limb of internal capsule L
Corona radiata	17.7%	Anterior corono radiata L
		Posterior corona radiata R
		Superior corona radiata L
		Superior corona radiata R
Cingulum (cingulate gyrus)	8.6%	Cingulum (cingulate gyrus) R
Superior fronto-occipital fasciculus	2.8%	Superior fronto-occipital fasciculus L
Posterior thalamic radiation	0.1%	Posterior thalamic radiation R

L = Left hemisphere, R = Right hemisphere.

#### 3.3.4 Subject-level longitudinal changes in altered WM mask

Repeated measures ANOVA and post hoc pairwise analysis of sessions confirmed that the altered white matter mask (ROI_ALT_) was associated with statistically-significant effects of session in both mean FA and mean MD for FBA, but not for NCA (see Table 6, Table 7). Comparisons in FBA with *In1* yielded the strongest observed effects, while the smallest session-wise changes were observed for *Post*.

**Table 6:** Repeated measures ANOVA outcomes evaluating mean FA and mean MD in ROI_ALT_ across indicated sessions.

Group	FA/MD	Sessions	*F*-statistic	Corrected–*p*
NCA	${\bar{\mathrm{FA}}}_{\mathrm{ALT}}$	All sessions	F(1, 14) = 0.21	0.651
	${\bar{\mathrm{MD}}}_{\mathrm{ALT}}$	All sessions	F(1, 14) = 0.48	0.504
FBA	${\bar{\mathrm{FA}}}_{\mathrm{ALT}}$	All sessions	F(3, 180) = 20.82	**<10**^−**4**^⁎
	${\bar{\mathrm{MD}}}_{\mathrm{ALT}}$	All sessions	F(3, 180) = 7.91	**<10**^−**4**^⁎

Where the sphericity assumption was violated, *p*-values were corrected with Huynh–Feldt's method.

⁎ Indicates a result significant at the (corrected) *p* < 0.05 level.

**Table 7:** Post hoc pairwise *t*-test comparisons of session-wise mean FA and mean MD measurements in ROI_ALT_ of FBA as acquired over all sessions, given a significant effect of session observed in Table 6).

Group	FA/MD	Pairwise comparisons	*t*-statistic	Corrected–*p*
FBA	${\bar{\mathrm{FA}}}_{\mathrm{ALT}}$	*In1* vs. *Pre*	7.84	**<10**^−**4**^⁎
		*In2* vs. *Pre*	4.10	**<10**^−**4**^⁎
		*Post* vs. *Pre*	3.19	**0.002**⁎
		*In2* vs. *In1*	−3.75	**<10**^−**3**^⁎
		*Post* vs. *In1*	−4.65	**<10**^−**4**^⁎
		*Post* vs. *In2*	−0.90	0.367
	${\bar{\mathrm{MD}}}_{\mathrm{ALT}}$	*In1* vs. *Pre*	−4.83	**<10**^−**4**^⁎
		*In2* vs. *Pre*	−2.60	**0.014**⁎
		*Post* vs. *Pre*	−1.97	0.050
		*In2* vs. *In1*	2.23	**0.027**⁎
		*Post* vs. *In1*	2.86	**0.007**⁎
		*Post* vs. *In2*	0.63	0.532

Duncan's method was used to correct for multiple comparisons. (Note that a positive sign for *t* indicates that the measurement in first listed session is greater than the measurement in the second.)

⁎ Indicates a result significant at the (corrected) *p* < 0.05 level.

**Fig. 11:**
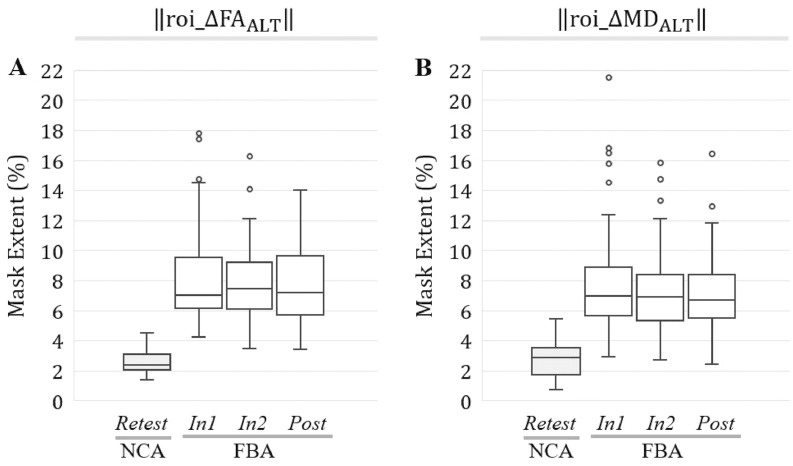
At all follow-up sessions, football athletes (FBA) exhibited significantly (*p* < 0.001) greater spatial extents of substantial changes in FA and MD, in ROI_ALT_, than did noncollision athletes (NCA). Box-and-whisker plots are presented at each follow-up session for (A) ‖roi_ΔFA_ALT,*j,i*_‖; (B) ‖roi_ΔMD_ALT,*j,i*_‖.

As observed for ROI_WM_, the spatial extents of substantial changes in both FA and MD within the altered white matter mask—‖roi_ΔFA_ALT,*j,i*_‖, and ‖roi_ΔMD_ALT,*j,i*_‖—were found to be significantly larger (*p* < 0.0001; *t*-test or Wilcoxon rank-sum test) at each follow-up session for FBA relative to NCA (Fig. 11).

**Fig. 12:**
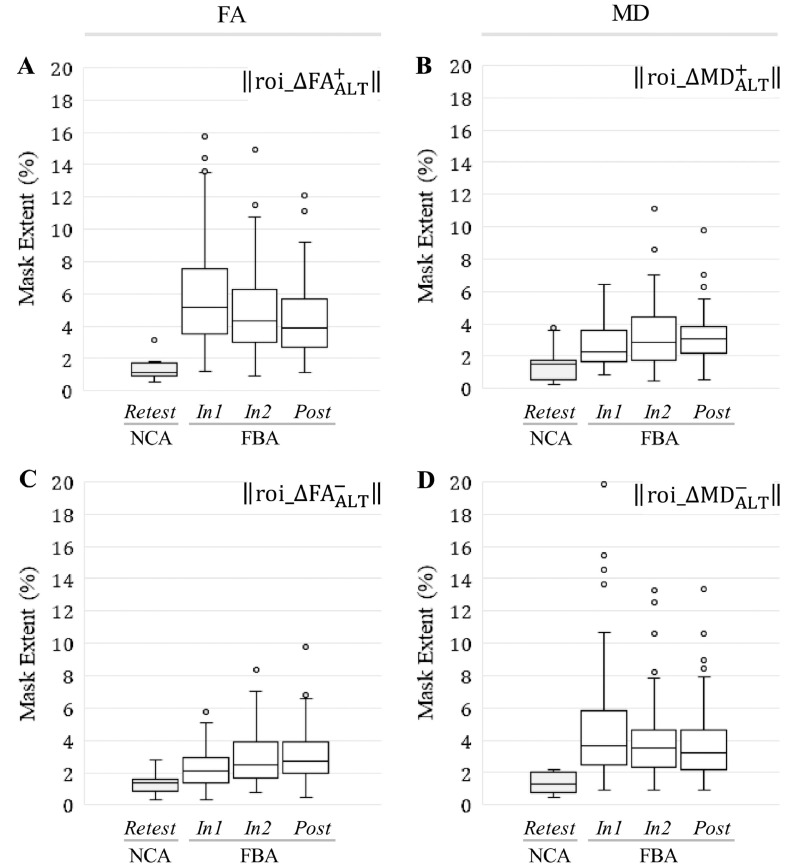
At all follow-up sessions, football athletes (FBA) exhibited significantly (*p* < 0.001) greater spatial extents of substantial increase/decrease in FA and MD, within ROI_ALT_, than did noncollision athletes (NCA). Box-and-whisker plots are presented at each follow-up session for (A) ‖$\text{roi}\_\Delta {\text{FA}}_{\text{ALT},j,i}^{+}$‖; (B) ‖$\text{roi}\_\Delta {\text{MD}}_{\text{ALT},j,i}^{+}$‖; (C) ‖$\text{roi}\_\Delta {\text{FA}}_{\text{ALT},j,i}^{-}$‖; (D) ‖$\text{roi}\_\Delta {\text{MD}}_{\text{ALT},j,i}^{-}$‖.

Similarly, all signed change masks associated with ROI_ALT_ were found to be significantly larger in spatial extent (*p* < 0.001; *t*-test or Wilcoxon rank-sum test) at each follow-up session for FBA, relative to NCA (Fig. 12).

Change mask spatial extents within ROI_ALT_ exhibited greater linkage to HAE exposure than for ROI_WM_. As seen in Table 8, correlations significant at the *p*_Bonferroni_ < 0.05 level were observed between accrual of HAEs (at thresholds of 20 g, 30 g, and 40 g) and the spatial extent of the signed change masks in the altered white matter.

**Table 8:** Pearson's and Spearman's correlation coefficients relating cumulative HAE counts exceeding the indicated minimum linear acceleration threshold (20–70 g), across all follow-up imaging sessions (i.e., *In1, In2, Post*; using the end-of-season HAE count for *Post*), to the spatial extents of the signed change masks at the corresponding times for ROI_ALT_.

ROI extent	‖$\text{roi}\_\Delta {\text{FA}}_{\text{ALT}}^{+}$‖		‖$\text{roi}\_\Delta {\text{FA}}_{\text{ALT}}^{-}$‖		‖$\text{roi}\_\Delta {\text{MD}}_{\text{ALT}}^{+}$‖		‖$\text{roi}\_\Delta {\text{MD}}_{\text{ALT}}^{-}$‖	
Included HAEs	Pearson	Spearman	Pearson	Spearman	Pearson	Spearman	Pearson	Spearman
PLA > 20 g	−0.088	−0.102	**0.291**⁎	**0.246**⁎	**0.261**⁎	**0.242**⁎	−0.088	−0.159
PLA > 30 g	−0.076	−0.112	**0.252**⁎	**0.222**⁎	**0.224**⁎	**0.227**⁎	−0.065	−0.172
PLA > 40 g	−0.049	−0.106	**0.204**⁎	**0.195**⁎	0.179	**0.210**⁎	−0.033	−0.168
PLA > 50 g	−0.043	−0.113	0.146	0.153	0.127	0.171	−0.035	−0.171
PLA > 60 g	−0.021	−0.071	0.080	0.083	0.295	0.110	−0.020	−0.123
PLA > 70 g	−0.018	−0.057	0.082	0.100	0.073	0.137	−0.021	−0.112

⁎ Correlation significant at the *p*_*Bonferroni*_ < 0.05 level.

**Fig. 13:**
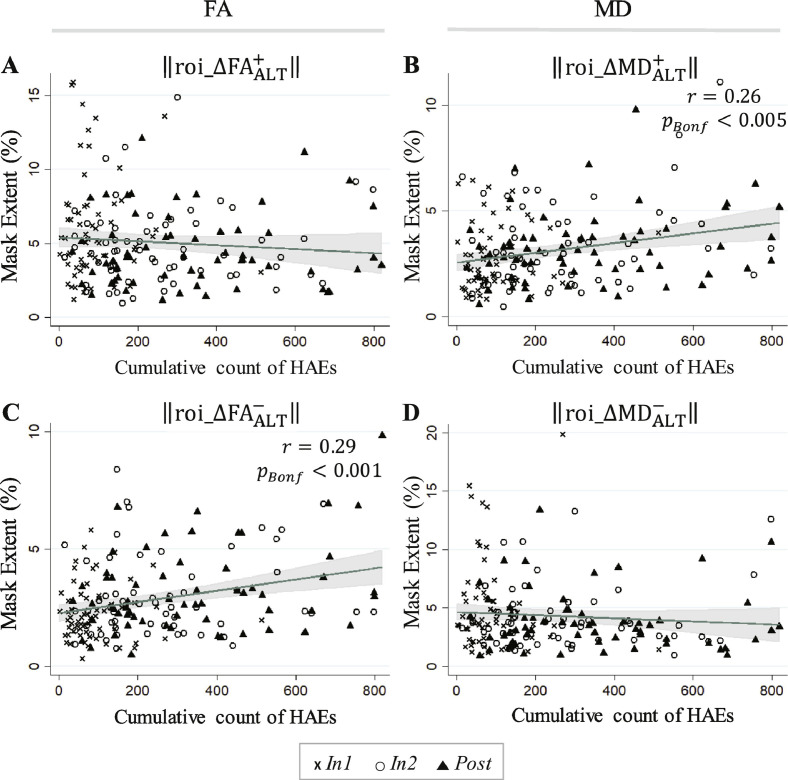
Linear predictions and associated confidence bands are superimposed on 183 FBA samples (61 subjects at each of three follow-up sessions) for (A) ‖$\text{roi}\_\Delta {\text{FA}}_{\text{ALT}}^{+}$‖; (B) ‖$\text{roi}\_\Delta {\text{MD}}_{\text{ALT}}^{+}$‖; (C) ‖$\text{roi}\_\Delta {\text{FA}}_{\text{ALT}}^{-}$‖; (D) ‖$\text{roi}\_\Delta {\text{MD}}_{\text{ALT}}^{-}$‖. Significant linear regressions (Bonferroni-corrected; see Table 8) were found in FBA for the spatial extent of the ROI_ALT_ signed change masks associated with (i) a decrease in FA ($\text{roi}\_\Delta {\text{FA}}_{\text{ALT}}^{-}$), and (ii) an increase in MD ($\text{roi}\_\Delta {\text{MD}}_{\text{ALT}}^{+}$), as a function of the cumulative count of HAEs exceeding 20 g. The white matter changes with which statistically-significant regressions were observed—increased MD and decreased FA—are typically associated with neural injury (e.g. Wieshmann et al., 1999, Arfanakis et al., 2002, Kraus et al., 2007, Mac Donald et al., 2007a, Mac Donald et al., 2007b; Shitaka et al., 2011, Magnoni et al., 2015, Sundman et al., 2015, Pan et al., 2016, Kantarci et al., 2017). The symbol *r* represents a Pearson's correlation coefficient.

Regressions against the HAE count at 20 g, the threshold associated with the most significant correlation with signed change mask spatial extents, are shown for all signed change masks in Fig. 13. Consistent with Table 8, the regression fits against the to-date accumulation of HAEs exceeding 20 g for ‖$\text{roi}\_\Delta {\text{FA}}_{\text{ALT}}^{-}$‖ and ‖$\text{roi}\_\Delta {\text{MD}}_{\text{ALT}}^{+}$‖ were found to be statistically significant.

## 4 Discussion

Retrospective examination of DWI data, collected longitudinally over single seasons of participation by male high school athletes, revealed that athletes who experience repetitive HAEs exhibit greater changes in white matter diffusivity than athletes who do not experience such HAEs, and that these white matter changes were significantly correlated with the longitudinal accumulation of exposure to HAEs. A novel regional analysis of signed changes in diffusion measures provided enhanced granularity of pathophysiology detection beyond that achieved through traditional whole-brain or regional averages. It facilitated identification of spatial extents of white matter in which FA and/or MD either increased or decreased to lie outside the “normal” range. Regions identified are similar to those found in previous studies of sports-related concussion (Cubon et al., 2011; Gajawelli et al., 2013; Lipton et al., 2013; McAllister et al., 2014; Pan et al., 2016; Mustafi et al., 2018). These change extents developed within the first 6 weeks of collision activity, and generally persisted into the post-season. Critically, the spatial extents of the white matter exhibiting those diffusivity changes normally associated with brain injury (decreased FA and increased MD) were found to exhibit statistically-significant correlations with cumulative HAE exposure. Such longitudinal changes, arising during and correlated with exposure to HAEs, support heightened public concern for athletes who participate in collision-based sports during periods of rapid brain development (Marar et al., 2012; Stamm et al., 2015).

### 4.1 Diffusion changes linked to HAE accumulation

This work expands on findings of previous studies of diffusion measures in athletes by examining both positive and negative alterations in FA and MD over the course of the season. Previous work has reported that these different directions of alteration are associated with different pathophysiology. As noted earlier, decreased FA and increased MD measurements are commonly linked to underlying disruption of tissue structure (Mac Donald et al., 2007a, Mac Donald et al., 2007b; Shitaka et al., 2011; Magnoni et al., 2015; Sundman et al., 2015; Pan et al., 2016; Kantarci et al., 2017), while increased FA and decreased MD measurements likely relate to altered axonal membrane health (Povlishock and Katz, 2005; Marmarou et al., 2006; Wilde et al., 2008; Chu et al., 2010; Bazarian et al., 2012).

Locations that exhibited a statistically-significant change, at any follow-up session relative to baseline, comprise a region of altered white matter overlapping with tracts commonly reported in previous work to be affected by mTBI or concussion. White matter regions exhibiting significant alteration in FA and/or MD (ROI_ALT_) at the group level were predominantly (53% of the volume) located in the corpus callosum (Arfanakis et al., 2002; Inglese et al., 2005; Bazarian et al., 2007, Bazarian et al., 2014; Kraus et al., 2007; Lipton et al., 2008; Wilde et al., 2008; Mayer et al., 2010, Mayer et al., 2012; Cubon et al., 2011; Koerte et al., 2012; Ling et al., 2012b; McAllister et al., 2012, McAllister et al., 2014; Gajawelli et al., 2013; Gong et al., 2018; Mustafi et al., 2018) and superior longitudinal fasciculus (Kraus et al., 2007; Cubon et al., 2011; Koerte et al., 2012; Mayer et al., 2012; Bazarian et al., 2014; Bahrami et al., 2016; Manning et al., 2017; Myer et al., 2018; Yuan et al., 2018).

From a biomechanical perspective, blows randomly incident on the head will exhibit a primary intersection of induced strain in the vicinity of the center of the brain (Corsellis et al., 1973; Gurdjian and Gurdjian, 1976; Ji et al., 2014). Therefore, centrally-located tracts are a priori expected to be at greater risk of accumulation of strain (and associated pathophysiology) from exposure to repeated HAEs arising from blows to the head or body.

Longitudinal changes in diffusion measures are unlikely to have arisen from either normal white matter development or differences in the level of physical activity across the groups, across assessment times. Given that these spatial extents were defined based on the test-retest variability of the NCA population over a time-window (average = 8 weeks) comparable with the inter-session period (average = 6 weeks) for the FBA, it is unlikely that the substantive changes observed in FBA from *Pre* to *In1* may be explained by normal development. Further, given that both groups are actively involved in conditioning activities at the time of their baseline sessions, these changes in FBA are less likely to be the consequence of altered levels of physical activity.

### 4.2 Implied HAE-induced damage accumulation

The signed change masks, focusing on the spatial extent of the brain exhibiting a particular direction of deviation in white matter diffusion, may help us explain why previous studies of FA and/or MD have obtained variable outcomes. Our findings suggest that we are observing a continuous white matter injury and repair process. This potential white matter tract injury (tissue damage) may begin with inflammation or axonal swelling (associated with increased FA) and progresses to axonal injury (associated with decreased FA).

Conversely, when examining only the mean effect on FA or MD, athletes early in the process may balance athletes in the later stages, and the mean observed alteration in diffusion measures would be expected to be close to zero. See Table 3 and Fig. 5, Fig. 6, in which the average change in mean FA over the entire white matter skeleton (i.e., ROI_WM_) would be expected to be slightly positive at *In1* and *In2*, but to approach zero at *Post*. The opposite trend is observed for mean MD. Thus, within an athlete, different magnitudes or signs of change in mean FA or mean MD could be observed, depending on the time since initiation of the injury and repair process. Given the rate at which athletes accumulate HAEs depends on a wide range of factors (e.g., position, playing time, style of play, competition level), a population assessment at the end of a season captures the averaged effects of this potential white matter injury process.

The overall hypothesis of an active injury and repair process is supported by our observation of multiple, sparsely-distributed locations in the white matter that exhibit either increases or decreases in FA and MD. When examined on a regional basis, these distributions generally led to changes in FA and MD that fluctuated between increases and decreases across sessions. Thus, some studies (e.g., McAllister et al., 2012; Lipton et al., 2013; Bahrami et al., 2016) may have captured post-season measures at a point when regions with increased FA may have balanced those regions exhibiting decreased FA (e.g., see *Post* sessions for FA-related mask spatial extents in Fig. 8). Therefore, this lack of mean change in diffusion measures may not imply the absence of underlying disturbances in axonal architecture or tissue injury.

### 4.3 Limitations

In a study of athletes involved in collision-based sports, it must also be acknowledged that participants may be exposed to HAEs outside the known times of practices and games, possibly through non-sanctioned play or involvement in other collision-based activities (e.g., club sports). Coupled with such additional potential exposure, complete knowledge of the use of anti-inflammatory drugs (e.g., aspirin, ibuprofen, naproxen) is unknown, and such medications could affect measurements of diffusion, particularly if the associated control of inflammation is variable across measurement sessions. Although the study incorporates an age-appropriate and gender-matched non-collision sport control population that adjusts for confounds such as environmental factors (Tan et al., 1998; Dechent et al., 1999; Babb et al., 2004) and exercise (Maddock et al., 2011), it would be desirable to achieve a greater balance of racial and ethnic categories across the subject populations. Further, individuals in this age bracket are experiencing many biological changes, including rapid growth of the brain (Giorgio et al., 2010; Lebel and Beaulieu, 2011; Lebel et al., 2012; Simmonds et al., 2014), for which collection of a longer-term longitudinal dataset for the NCA population would facilitate more powerful parallel comparisons.

From a technical perspective, the exact relationship between diffusion MRI markers of white matter and underlying tissue damage is still a matter of debate. Myelin and cellular membranes each play a role in restricting water diffusion in the nervous system (Barkovich, 2000; Lancaster et al., 2003; Sotak, 2004), with cellular membranes creating boundaries between water pools of different mobility. Thus, there may not exist a strict one-to-one relationship between a given structural alteration and a particular MR measure. For example, FA may increase as a result of restricted axial diffusivity, facilitated parallel diffusivity, or some combination of the two. In addition, the use of traditional, single *b*-value diffusion weighted imaging is partially limiting (e.g., to correct susceptibility distortions).

We note that the location of significantly altered white matter is unexpectedly structured, running from the left frontal lobe through the corpus callosum to the right parietal lobe. In theory, one might expect such a region to be more uniformly distributed if the athletes are experiencing an unbiased distribution of impacts to the head. Given that the HAE monitoring devices used in this study have not been shown to provide meaningful source location information, we cannot effectively assess whether the incoming blows were uniformly distributed or biased toward one side.

Complex white matter structures (e.g., crossing fibers) will potentially invalidate the assumption of a primary orientation of fibers, which is normally assumed to be represented by the diffusion tensor's main eigenvector (Mori and Tournier, 2013). A longitudinal approach that applies a more powerful technique such as diffusion kurtosis imaging (e.g., Davenport et al., 2016) could provide additional insight.

## 5 Conclusion

This study documents that changes in diffusivity of white matter in high school-aged athletes participating in American football are correlated with accumulation of HAEs throughout the course of a season of participation. This correlation provides evidence of a potential mechanically-initiated white matter injury and repair process. Such a process is consistent with evidence from previous work involving both neuroimaging and HAE monitoring (e.g., Breedlove et al., 2012; Lipton et al., 2013; Bazarian et al., 2014; Davenport et al., 2014, Davenport et al., 2016; Bahrami et al., 2016; Svaldi et al., 2018; Bari et al., 2018) suggesting deviations of measures of brain structure and function are correlated with aggregate HAE exposure. While there may be a threshold beyond which physiologic function is acutely altered (Svaldi et al., 2018; Bari et al., 2018), this study demonstrates that the potential damage accumulation is more gradual and represents the cumulative effect of possibly all HAE exposures. Future effort should be directed at reducing the number and magnitude of events experienced by collision-sport athletes, whether through enhanced protective equipment, improved technique instruction, or modification of rules.

The following are the supplementary data related to this article.

Supplementary Video 1 A video visualizing the group-level altered white matter region. This rotating brain shows projection images shown in Fig. 10 more effectively. Supplementary Video 1

## References

[bb0005] Abbas K., Shenk T.E., Poole V.N., Breedlove E.L., Leverenz L.J., Nauman E.A., Talavage T.M., Robinson M.E. (2015). Alteration of default mode network in high school football athletes due to repetitive subconcussive mild traumatic brain injury: a resting-state functional magnetic resonance imaging study. Brain Connect..

[bb0010] Abbas K., Shenk T.E., Poole V.N., Robinson M.E., Leverenz L.J., Nauman E.A., Talavage T.M. (2015). Effects of repetitive sub-concussive brain injury on the functional connectivity of default mode network in high school football athletes. Dev. Neuropsychol..

[bb0015] Andersson J.L., Sotiropoulos S.N. (2016). An integrated approach to correction for off-resonance effects and subject movement in diffusion MR imaging. Neuroimage.

[bb0020] Andersson J.L.R., Jenkinson M., Smith S. (2007). Non-linear registration aka spatial normalisation FMRIB Technial report TR07JA2. Tech. Rep.

[bb0025] Andersson J.L.R., Jenkinson M., Smith S.M. (2007). Non-linear optimisation. FMRIB technical report TR07JA1. Tech. Rep. June.

[bb0030] Andersson J.L., Graham M.S., Zsoldos E., Sotiropoulos S.N. (2016). Incorporating outlier detection and replacement into a non-parametric framework for movement and distortion correction of diffusion MR images. Neuroimage.

[bb0035] Arfanakis K., Haughton V.M., Carew J.D., Rogers B.P., Dempsey R.J., Meyerand E. (2002). Diffusion tensor MR imaging in diffuse axonal injury. Am. J. Neuroradiol..

[bb0040] Babb S.M., Ke Y., Lange N., Kaufman M.J., Renshaw P.F., Cohen B.M. (2004). Oral choline increases choline metabolites in human brain. Psychiatry Res. Neuroimaging.

[bb0045] Bahrami N., Sharma D., Rosenthal S., Davenport E.M., Urban J.E., Wagner B., Jung Y., Vaughan C.G., Gioia G.A., Stitzel J.D., Whitlow C.T., Maldjian J.A. (Dec. 2016). Subconcussive head impact exposure and white matter tract changes over a single season of youth football. Radiology.

[bb0050] Bailes J.E., Petraglia A.L., Omalu B.I., Nauman E., Talavage T. (Nov. 2013). Role of subconcussion in repetitive mild traumatic brain injury. J. Neurosurg..

[bb0055] Bari S., Svaldi D.O., Jang I., Shenk T.E., Poole V.N., Lee T., Dydak U., Rispoli J.V., Nauman E.A., Talavage T.M. (2018). Dependence on subconcussive impacts of brain metabolism in collision sport athletes: an MR spectroscopic study. Brain Imaging Behav..

[bb0060] Barkovich J.A. (2000). Concepts of myelin and myelination in neuroradiology. Am. J. Neuroradiol..

[bb0065] Bazarian J.J., Zhong J., Blyth B., Zhu T., Kavcic V., Peterson D. (2007). Diffusion tensor imaging detects clinically important axonal damage after mild traumatic brain injury: a pilot study. J. Neurotrauma.

[bb0070] Bazarian J.J., Zhu T., Blyth B., Borrino A., Zhong J. (2012). Subject-specific changes in brain white matter on diffusion tensor imaging after sports-related concussion. Magn. Reson. Imaging.

[bb0075] Bazarian J.J., Zhu T., Zhong J., Janigro D., Rozen E., Roberts A., Javien H., Merchant-Borna K., Abar B., Blackman E.G. (2014). Persistent, long-term cerebral white matter changes after sports-related repetitive head impacts. PLoS One.

[bb0080] Beaulieu C. (2002). The basis of anisotropic water diffusion in the nervous system – a technical review. NMR Biomed..

[bb0085] Behrens T., Woolrich M., Jenkinson M., Johansen-Berg H., Nunes R., Clare S., Matthews P., Brady J., Smith S. (2003). Characterization and propagation of uncertainty in diffusion-weighted MR imaging. Magn. Reson. Med..

[bb0090] Breedlove E.L., Robinson M., Talavage T.M., Morigaki K.E., Yoruk U., O'Keefe K., King J., Leverenz L.J., Gilger J.W., Nauman E.A. (2012). Biomechanical correlates of symptomatic and asymptomatic neurophysiological impairment in high school football. J. Biomech..

[bb0095] Broglio S.P., Sosnoff J.J., Rosengren K.S., McShane K. (2009). A comparison of balance performance: computerized dynamic posturography and a random motion platform. Arch. Phys. Med. Rehabil..

[bb0100] Christman C.W., Grady M.S., Walker S.A., Holloway K.L., Povlishock J.T. (1994). Ultrastructural studies of diffuse axonal injury in humans. J. Neurotrauma.

[bb0105] Chu Z., Wilde E.A., Hunter J.V., McCauley S.R., Bigler E.D., Troyanskaya M., Yallampalli R., Chia J.M., Levin H.S. (2010). Voxel-based analysis of diffusion tensor imaging in mild traumatic brain injury in adolescents. AJNR Am. J. Neuroradiol..

[bb0110] Chun I.Y., Mao X., Breedlove E.L., Leverenz L.J., Nauman E.A., Talavage T.M. (2015). DTI detection of longitudinal WM abnormalities due to accumulated head impacts. Dev. Neuropsychol..

[bb0115] Corsellis J.A., Bruton C.J., Freeman-Browne D. (1973). The aftermath of boxing. Psychol. Med..

[bb0120] Cubon V.A., Putukian M., Boyer C., Dettwiler A. (2011). A diffusion tensor imaging study on the white matter skeleton in individuals with sports-related concussion. J. Neurotrauma.

[bb0125] Cummiskey B., Schiffmiller D., Talavage T.M., Leverenz L., Meyer J.J., Adams D., Nauman E.A. (2017). Reliability and accuracy of helmet-mounted and head-mounted devices used to measure head accelerations. Proc. Inst. Mech. Eng. P J. Sports Eng. Technol..

[bb0130] Davenport E.M., Whitlow C.T., Urban J.E., Espeland M.A., Jung Y., Rosenbaum D.A., Gioia G.A., Powers A.K., Stitzel J.D., Maldjian J.A. (2014). Abnormal white matter integrity related to head impact exposure in a season of high school varsity football. J. Neurotrauma.

[bb0135] Davenport E.M., Apkarian K., Whitlow C.T., Urban J.E., Jensen J.H., Szuch E., Espeland M.A., Jung Y., Rosenbaum D.A., Gioia G.A., Powers A.K., Stitzel J.D., Maldjian J.A. (Dec. 2016). Abnormalities in diffusional kurtosis metrics related to head impact exposure in a season of high school varsity football. J. Neurotrauma.

[bb0140] Dechent P., Pouwels P.J.W., Wilken B., Hanefeld F., Frahm J. (1999). Increase of total creatine in human brain after oral supplementation of creatine-monohydrate. Am. J. Phys. Regul. Integr. Comp. Phys..

[bb0145] Gajawelli N., Lao Y., Apuzzo M.L., Romano R., Liu C., Tsao S., Hwang D., Wilkins B., Lepore N., Law M. (2013). Neuroimaging changes in the brain in contact versus noncontact sport athletes using diffusion tensor imaging. World Neurosurg..

[bb0150] Giorgio A., Watkins K., Chadwick M., James S., Winmill L., Douaud G., De Stefano N., Matthews P., Smith S., Johansen-Berg H., James A. (2010). Longitudinal changes in grey and white matter during adolescence. Neuroimage.

[bb0155] Giza C.C., Hovda D.A. (2001). The neurometabolic cascade of concussion. J. Athl. Train..

[bb0160] Giza C.C., Hovda D.A. (2014). The new neurometabolic cascade of concussion. Neurosurgery.

[bb0165] Giza C.C., Prins M.L. (2006). Is being plastic fantastic? Mechanisms of altered plasticity after developmental traumatic brain injury. Dev. Neurosci..

[bb0170] Giza C.C., Prins M.L., Hovda D.A. (2017). Its not all fun and games: sports, concussions, and neuroscience. Neuron.

[bb0175] Glantz S.A. (2012). Primer of Biostatistics.

[bb0180] Gong N.-J., Kuzminski S., Clark M., Fraser M., Sundman M., Guskiewicz K., Petrella J.R., Liu C. (2018). Microstructural alterations of cortical and deep gray matter over a season of high school football revealed by diffusion kurtosis imaging. Neurobiol. Dis..

[bb0185] Grady M.S., Mclaughlin M.R., Christman C.W., Valadka A.B., Fligner C.L., Povlishock J.T. (Mar. 1993). The use of antibodies targeted against the neurofilament subunits for the detection of diffuse axonal injury in humans. J. Neuropathol. Exp. Neurol..

[bb0190] Gurdjian E.S., Gurdjian E.S. (1976). Cerebral contusions: re-evaluation of the mechanism of their development. J. Trauma Inj. Infec. Crit. Care.

[bb0195] Henry L.C., Tremblay J., Tremblay S., Lee A., Brun C., Lepore N., Theoret H., Ellemberg D., Lassonde M. (2011). Acute and chronic changes in diffusivity measures after sports concussion. J. Neurotrauma.

[bb0200] Hua Li H., Lee S.M., Cai Y., Sutton R.L., Hovda D.A. (2004). Differential gene expression in *Hippocampus* following experimental brain trauma reveals distinct features of moderate and severe injuries. J. Neurotrauma.

[bb0205] Inglese M., Makani S., Johnson G., Cohen B.A., Silver J.A., Gonen O., Grossman R.I. (2005). Diffuse axonal injury in mild traumatic brain injury: a diffusion tensor imaging study. J. Neurosurg..

[bb0210] Jadischke R., Viano D.C., Dau N., King A.I., McCarthy J. (2013). On the accuracy of the head impact telemetry (HIT) system used in football helmets. J. Biomech..

[bb0215] Jenkinson M., Beckmann C.F., Behrens T.E.J., Woolrich M.W., Smith S.M. (2012). FSL. Neuroimage.

[bb0220] Ji S., Ghadyani H., Bolander R.P., Beckwith J.G., Ford J.C., McAllister T.W., Flashman L.A., Paulsen K.D., Ernstrom K., Jain S., Raman R., Zhang L., Greenwald R.M. (2014). Parametric comparisons of intracranial mechanical responses from three validated finite element models of the human head. Ann. Biomed. Eng..

[bb0225] Johnson V., Stewart W., Smith D. (2013). Axonal pathology in traumatic brain injury. Exp. Neurol..

[bb0230] Johnson B., Neuberger T., Gay M., Hallett M., Slobounov S. (Dec. 2014). Effects of subconcussive head trauma on the default mode network of the brain. J. Neurotrauma.

[bb0235] Kantarci K., Murray M.E., Schwarz C.G., Reid R.I., Przybelski S.A., Lesnick T., Zuk S.M., Raman M.R., Senjem M.L., Gunter J.L., Boeve B.F., Knopman D.S., Parisi J.E., Petersen R.C., Jack C.R., Dickson D.W. (2017). White-matter integrity on DTI and the pathologic staging of Alzheimer's disease. Neurobiol. Aging.

[bb0240] Koerte I.K., Kaufmann D., Hartl E., Bouix S., Pasternak O., Kubicki M., Rauscher A., Li D.K.B., Dadachanji S.B., Taunton J.A., Forwell L.A., Johnson A.M., Echlin P.S., Shenton M.E. (2012). A prospective study of physician-observed concussion during a varsity university hockey season: white matter integrity in ice hockey players. Part 3 of 4. Neurosurg. Focus..

[bb0245] Kraus M.F., Susmaras T., Caughlin B.P., Walker C.J., Sweeney J.A., Little D.M. (2007). White matter integrity and cognition in chronic traumatic brain injury: a diffusion tensor imaging study. Brain.

[bb0250] Kuzminski S., Clark M., Fraser M., Haswell C., Morey R., Liu C., Choudhury K., Guskiewicz K., Petrella J. (2018). White matter changes related to subconcussive impact frequency during a single season of high school football. Am. J. Neuroradiol..

[bb0255] Lancaster J.L., Andrews T., Hardies L.J., Dodd S., Fox P.T. (2003). Three-pool model of white matter. J. Magn. Reson. Imaging.

[bb0260] Lebel C., Beaulieu C. (2011). Longitudinal development of human brain wiring continues from childhood into adulthood. J. Neurosci..

[bb0265] Lebel C., Gee M., Camicioli R., Wieler M., Martin W., Beaulieu C. (2012). Diffusion tensor imaging of white matter tract evolution over the lifespan. Neuroimage.

[bb0270] Lehman E.J., Hein M.J., Baron S.L., Gersic C.M. (2012). Neurodegenerative causes of death among retired National Football League players. Neurology.

[bb0275] Ling J., Merideth F., Caprihan A., Pena A., Teshiba T., Mayer A.R. (2012). Head injury or head motion? Assessment and quantification of motion artifacts in diffusion tensor imaging studies. Hum. Brain Mapp..

[bb0280] Ling J.M., Peña A., Yeo R.A., Merideth F.L., Klimaj S., Gasparovic C., Mayer A.R. (2012). Biomarkers of increased diffusion anisotropy in semi-acute mild traumatic brain injury: a longitudinal perspective. Brain.

[bb0285] Lipton M.L., Gellella E., Lo C., Gold T., Ardekani B.A., Shifteh K., Bello J.A., Branch C.A. (2008). Multifocal white matter ultrastructural abnormalities in mild traumatic brain injury with cognitive disability: a voxel-wise analysis of diffusion tensor imaging. J. Neurotrauma.

[bb0290] Lipton M.L., Kim N., Zimmerman M.E., Kim M., Stewart W.F., Branch C.A., Lipton R.B. (2013). Soccer heading is associated with white matter microstructural and cognitive abnormalities. Radiology.

[bb0295] Loane D.J., Byrnes K.R. (2010). Role of microglia in neurotrauma. Neurotherapeutics.

[bb0300] Mac Donald C., Dikranian K., Song S., Bayly P., Holtzman D., Brody D. (2007). Detection of traumatic axonal injury with diffusion tensor imaging in a mouse model of traumatic brain injury. Exp. Neurol..

[bb0305] Mac Donald C.L., Dikranian K., Bayly P., Holtzman D., Brody D. (2007). Diffusion tensor imaging reliably detects experimental traumatic axonal injury and indicates approximate time of injury. J. Neurosci..

[bb0310] Maddock R.J., Casazza G.A., Buonocore M.H., Tanase C. (2011). Vigorous exercise increases brain lactate and Glx (glutamate + glutamine): a dynamic 1H-MRS study. Neuroimage.

[bb0315] Magnoni S., Mac Donald C.L., Esparza T.J., Conte V., Sorrell J., Macrì M., Bertani G., Biffi R., Costa A., Sammons B., Snyder A.Z., Shimony J.S., Triulzi F., Stocchetti N., Brody D.L. (2015). Quantitative assessments of traumatic axonal injury in human brain: concordance of microdialysis and advanced MRI. Brain.

[bb0320] Manning K.Y., Schranz A., Bartha R., Dekaban G.A., Barreira C., Brown A., Fischer L., Asem K., Doherty T.J., Fraser D.D., Holmes J., Menon R.S. (2017). Multiparametric MRI changes persist beyond recovery in concussed adolescent hockey players. Neurology.

[bb0325] Marar M., McIlvain N.M., Fields S.K., Comstock R.D. (2012). Epidemiology of concussions among United States high school athletes in 20 sports. Am. J. Sports Med..

[bb0330] Marmarou A., Signoretti S., Fatouros P.P., Portella G., Aygok G.A., Bullock M.R. (2006). Predominance of cellular edema in traumatic brain swelling in patients with severe head injuries. J. Neurosurg..

[bb0335] Mayer A.R., Ling J., Mannell M.V., Gasparovic C., Phillips J.P., Doezema D., Reichard R., Yeo R.A. (2010). A prospective diffusion tensor imaging study in mild traumatic brain injury. Neurology.

[bb0340] Mayer A.R., Ling J.M., Yang Z., Pena A., Yeo R.A., Klimaj S. (2012). Diffusion abnormalities in pediatric mild traumatic brain injury. J. Neurosci..

[bb0345] McAllister T.W., Ford J.C., Ji S., Beckwith J.G., Flashman L.A., Paulsen K., Greenwald R.M. (2012). Maximum principal strain and strain rate associated with concussion diagnosis correlates with changes in corpus callosum white matter indices. Ann. Biomed. Eng..

[bb0350] McAllister T.W., Ford J.C., Flashman L.A., Maerlender A., Greenwald R.M., Beckwith J.G., Bolander R.P., Tosteson T.D., Turco J.H., Raman R., Jain S. (2014). Effect of head impacts on diffusivity measures in a cohort of collegiate contact sport athletes. Neurology.

[bb0355] McCuen E., Svaldi D., Breedlove K., Kraz N., Cummiskey B., Breedlove E.L., Traver J., Desmond K.F., Hannemann R.E., Zanath E., Guerra A., Leverenz L., Talavage T.M., Nauman E.A. (2015). Collegiate women's soccer players suffer greater cumulative head impacts than their high school counterparts. J. Biomech..

[bb0360] McKee A.C., Cantu R.C., Nowinski C.J., Hedley-Whyte E.T., Gavett B.E., Budson A.E., Santini V.E., Lee H.-S., Kubilus C.A., Stern R.A. (2009). Chronic traumatic encephalopathy in athletes: progressive tauopathy after repetitive head injury. J. Neuropathol. Exp. Neurol..

[bb0365] Meier T.B., Brummel B.J., Singh R., Nerio C.J., Polanski D.W., Bellgowan P.S. (2015). The underreporting of self-reported symptoms following sports-related concussion. J. Sci. Med. Sport.

[bb0370] Miles L., Grossman R.I., Johnson G., Babb J.S., Diller L., Inglese M. (2008). Short-term DTI predictors of cognitive dysfunction in mild traumatic brain injury. Brain Inj..

[bb0375] Mori S., Tournier J.-D. (2013). Introduction to Diffusion Tensor Imaging: And Higher Order Models.

[bb0380] Mori S., Wakana S., Van Zijl P.C., Nagae-Poetscher L.M. (2005). MRI Atlas of Human White Matter.

[bb0385] Murugavel M., Cubon V., Putukian M., Echemendia R., Cabrera J., Osherson D., Dettwiler A. (2014). A longitudinal diffusion tensor imaging study assessing white matter fiber tracts after sports-related concussion. J. Neurotrauma.

[bb0390] Mustafi S.M., Harezlak J., Koch K.M., Nencka A.S., Meier T.B., West J.D., Giza C.C., DiFiori J.P., Guskiewicz K.M., Mihalik J.P., LaConte S.M., Duma S.M., Broglio S.P., Saykin A.J., McCrea M., McAllister T.W., Wu Y.-C. (2018). Acute white-matter abnormalities in sports-related concussion: a diffusion tensor imaging study from the NCAA-DoD CARE consortium. J. Neurotrauma.

[bb0395] Myer G.D., Yuan W., Barber Foss K.D., Smith D., Altaye M., Reches A., Leach J., Kiefer A.W., Khoury J.C., Weiss M., Thomas S., Dicesare C., Adams J., Gubanich P.J., Geva A., Clark J.F., Meehan W.P., Mihalik J.P., Krueger D. (2016). The effects of external jugular compression applied during head impact exposure on longitudinal changes in brain neuroanatomical and neurophysiological biomarkers: a preliminary investigation. Front. Neurol..

[bb0400] Myer G.D., Yuan W., Foss K.D.B., Thomas S., Smith D., Leach J., Kiefer A.W., Dicesare C., Adams J., Gubanich P.J., Kitchen K., Schneider D.K., Braswell D., Krueger D., Altaye M. (2016). Analysis of head impact exposure and brain microstructure response in a season-long application of a jugular vein compression collar: a prospective, neuroimaging investigation in American football. Br. J. Sports Med..

[bb0405] Myer G.D., Barber Foss K., Thomas S., Galloway R., Dicesare C.A., Dudley J., Gadd B., Leach J., Smith D., Gubanich P., Meehan W.P., Altaye M., Lavin P., Yuan W. (2018). Altered brain microstructure in association with repetitive subconcussive head impacts and the potential protective effect of jugular vein compression: a longitudinal study of female soccer athletes. Br. J. Sports Med..

[bb0410] Omalu B.I., DeKosky S.T., Hamilton R.L., Minster R.L., Kamboh M.I., Shakir A.M., Wecht C.H. (2006). Chronic traumatic encephalopathy in a national football league player. Neurosurgery.

[bb0415] Oni M.B., Wilde E.A., Bigler E.D., McCauley S.R., Wu T.C., Yallampalli R., Chu Z., Li X., Hunter J.V., Vasquez A.C., Levin H.S. (2010). Diffusion tensor imaging analysis of frontal lobes in pediatric traumatic brain injury. J. Child Neurol..

[bb0420] Pan J., Connolly I.D., Dangelmajer S., Kintzing J., Ho A.L., Grant G. (2016). Sports-related brain injuries: connecting pathology to diagnosis. Neurosurg. Focus..

[bb0425] Poole V.N., Abbas K., Shenk T.E., Breedlove E.L., Breedlove K.M., Robinson M.E., Leverenz L.J., Nauman E.A., Talavage T.M., Dydak U. (2014). MR spectroscopic evidence of brain injury in the non-diagnosed collision sport athlete. Dev. Neuropsychol..

[bb0430] Poole V.N., Breedlove E.L., Shenk T.E., Abbas K., Robinson M.E., Leverenz L.J., Nauman E.A., Dydak U., Talavage T.M. (2015). Sub-concussive hit characteristics predict deviant brain metabolism in football athletes. Dev. Neuropsychol..

[bb0435] Povlishock J.T., Katz D.I. (2005). Update of neuropathology and neurological recovery after traumatic brain injury. J. Head Trauma Rehabil..

[bb0440] Povlishock J.T., Becker D.P., Cheng C.L.Y., Vaughan G.W. (1983). Axonal change in minor head injury. J. Neuropathol. Exp. Neurol..

[bb0445] Robinson M.E., Lindemer E.R., Fonda J.R., Milberg W.P., McGlinchey R.E., Salat D.H. (2015). Close-range blast exposure is associated with altered functional connectivity in veterans independent of concussion symptoms at time of exposure. Hum. Brain Mapp..

[bb0450] Shenk T.E., Robinson M.E., Svaldi D.O., Abbas K., Breedlove K.M., Leverenz L.J., Nauman E.A., Talavage T.M. (2015). fMRI of visual working memory in high school football players. Dev. Neuropsychol..

[bb0455] Shitaka Y., Tran H.T., Bennett R.E., Sanchez L., Levy M.A., Dikranian K., Brody D.L. (2011). Repetitive closed-skull traumatic brain injury in mice causes persistent multifocal axonal injury and microglial reactivity. J. Neuropathol. Exp. Neurol..

[bb0460] Shultz S.R., MacFabe D.F., Foley K.A., Taylor R., Cain D.P. (2012). Sub-concussive brain injury in the long-evans rat induces acute neuroinflammation in the absence of behavioral impairments. Behav. Brain Res..

[bb0465] Simmonds D.J., Hallquist M.N., Asato M., Luna B. (2014). Developmental stages and sex differences of white matter and behavioral development through adolescence: a longitudinal diffusion tensor imaging (DTI) study. Neuroimage.

[bb0470] Slobounov S.M., Walter A., Breiter H.C., Zhu D.C., Bai X., Bream T., Seidenberg P., Mao X., Johnson B., Talavage T.M. (2017). The effect of repetitive subconcussive collisions on brain integrity in collegiate football players over a single football season: a multi-modal neuroimaging study. Neuroimage Clin..

[bb0475] Smith S.M. (2002). Fast robust automated brain extraction. Hum. Brain Mapp..

[bb0480] Smith S.M., Nichols T.E. (2009). Threshold-free cluster enhancement: addressing problems of smoothing, threshold dependence and localisation in cluster inference. Neuroimage.

[bb0485] Smith S.M., Jenkinson M., Woolrich M.W., Beckmann C.F., Behrens T.E., Johansen-Berg H., Bannister P.R., De Luca M., Drobnjak I., Flitney D.E., Niazy R.K., Saunders J., Vickers J., Zhang Y., De Stefano N., Brady J.M., Matthews P.M. (2004). Advances in functional and structural MR image analysis and implementation as FSL. Neuroimage.

[bb0490] Smith S.M., Jenkinson M., Johansen-Berg H., Rueckert D., Nichols T.E., Mackay C.E., Watkins K.E., Ciccarelli O., Cader M.Z., Matthews P.M., Behrens T.E. (2006). Tract-based spatial statistics: voxelwise analysis of multi-subject diffusion data. Neuroimage.

[bb0495] Sotak C.H. (2004). Nuclear magnetic resonance (NMR) measurement of the apparent diffusion coefficient (ADC) of tissue water and its relationship to cell volume changes in pathological states. Neurochem. Int..

[bb0500] Stamm J.M., Bourlas A.P., Baugh C.M., Fritts N.G., Daneshvar D.H., Martin B.M., McClean M.D., Tripodis Y., Stern R.A. (2015). Age of first exposure to football and later-life cognitive impairment in former NFL players. Neurology.

[bb0505] Stern R.A., Riley D.O., Daneshvar D.H., Nowinski C.J., Cantu R.C., McKee A.C. (2011). Long-term consequences of repetitive brain trauma: chronic traumatic encephalopathy. PM R.

[bb0510] Sundman M., Doraiswamy P.M., Morey R.A. (Sep. 2015). Neuroimaging assessment of early and late neurobiological sequelae of traumatic brain injury: implications for CTE. Front. Neurosci..

[bb0515] Svaldi D.O., Joshi C., Robinson M.E., Shenk T.E., Abbas K., Nauman E.A., Leverenz L.J., Talavage T.M. (2015). Cerebrovascular reactivity alterations in asymptomatic high school football players. Dev. Neuropsychol..

[bb0520] Svaldi D.O., McCuen E.C., Joshi C., Robinson M.E., Nho Y., Hannemann R., Nauman E.A., Leverenz L.J., Talavage T.M. (2017). Cerebrovascular reactivity changes in asymptomatic female athletes attributable to high school soccer participation. Brain Imaging Behav..

[bb0525] Svaldi D.O., Joshi C., McCuen E.C., Music J.P., Hannemann R., Leverenz L.J., Nauman E.A., Talavage T.M. (2018). Accumulation of high magnitude acceleration events predicts cerebrovascular reactivity changes in female high school soccer athletes. Brain Imaging Behav..

[bb0530] Talavage T.M., Nauman E.A., Breedlove E.L., Yoruk U., Dye A.E., Morigaki K.E., Feuer H., Leverenz L.J. (2014). Functionally-detected cognitive impairment in high school football players without clinically-diagnosed concussion. J. Neurotrauma.

[bb0535] Talavage T.M., Nauman E.A., Leverenz L.J. (2016). The role of medical imaging in the Recharacterization of mild traumatic brain injury using youth sports as a laboratory. Front. Neurol..

[bb0540] Tan J., Bluml S., Hoang T., Dubowitz D., Mevenkamp G., Ross B. (1998). Lack of effect of oral choline supplement on the concentrations of choline metabolites in human brain. Magn. Reson. Med..

[bb0545] Wieshmann U.C., Clark C.A., Symms M.R., Franconi F., Barker G.J., Shorvon S.D. (1999). Reduced anisotropy of water diffusion in structural cerebral abnormalities demonstrated with diffusion tensor imaging. Magn. Reson. Imaging.

[bb0550] Wilde E.A., Mccauley S.R., Hunter J.V., Bigler E.D., Chu Z., Wang Z.J., Hanten G.R., Troyanskaya M., Yallampalli R., Li B.X., Chia J., Levin H.S. (2008). Diffusion tensor imaging of acute mild traumatic brain injury in adolescents. Neurology.

[bb0555] Woolrich M.W., Jbabdi S., Patenaude B., Chappell M., Makni S., Behrens T., Beckmann C., Jenkinson M., Smith S.M. (2009). Bayesian analysis of neuroimaging data in FSL. Neuroimage.

[bb0560] Yuan W., Barber Foss K.D., Thomas S., DiCesare C.A., Dudley J.A., Kitchen K., Gadd B., Leach J.L., Smith D., Altaye M., Gubanich P., Galloway R.T., McCrory P., Bailes J.E., Mannix R., Meehan W.P., Myer G.D. (2018). White matter alterations over the course of two consecutive high-school football seasons and the effect of a jugular compression collar: a preliminary longitudinal diffusion tensor imaging study. Hum. Brain Mapp..

