# Supplementary figures and images for: Every hit matters: White matter diffusivity changes in high school football athletes are correlated with repetitive head acceleration event exposure
Source: Neuroimage Clin. 2019 Jul 16;24:101930. doi: 10.1016/j.nicl.2019.101930 (PMC6807364; doi:10.1016/j.nicl.2019.101930)

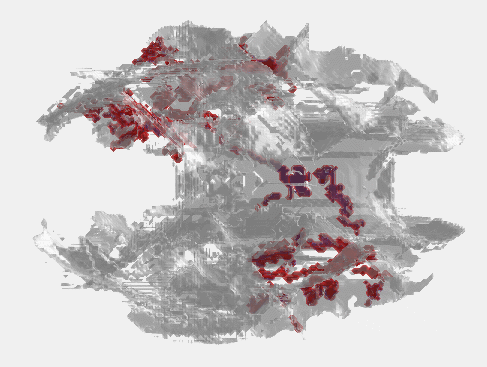

Supplement: Supplementary Video 1 — A video visualizing the group-level altered white matter region. This rotating brain shows projection images shown in Fig. 10 more effectively. [file mmc1.zip › mmc1.gif]
